# SARS-CoV-2 spike protein S1 subunit induces pro-inflammatory responses via toll-like receptor 4 signaling in murine and human macrophages

**DOI:** 10.1016/j.heliyon.2021.e06187

**Published:** 2021-02-02

**Authors:** Ken Shirato, Takako Kizaki

**Affiliations:** Department of Molecular Predictive Medicine and Sport Science, Kyorin University School of Medicine, 6-20-2 Shinkawa, Mitaka, Tokyo 181-8611, Japan

**Keywords:** SARS-CoV-2, Spike protein, S1 subunit, Toll-like receptor 4, Inflammation, Macrophage

## Abstract

Coronavirus disease 2019 (COVID-19), an infectious disease caused by severe acute respiratory syndrome coronavirus 2 (SARS-CoV-2), has now spread globally. Some patients develop severe complications including multiple organ failure. It has been suggested that excessive inflammation associated with the disease plays major role in the severity and mortality of COVID-19. To elucidate the inflammatory mechanisms involved in COVID-19, we examined the effects of SARS-CoV-2 spike protein S1 subunit (hereafter S1) on the pro-inflammatory responses in murine and human macrophages. Murine peritoneal exudate macrophages produced pro-inflammatory mediators in response to S1 exposure. Exposure to S1 also activated nuclear factor-κB (NF-κB) and c-Jun N-terminal kinase (JNK) signaling pathways. Pro-inflammatory cytokine induction by S1 was suppressed by selective inhibitors of NF-κB and JNK pathways. Treatment of murine peritoneal exudate macrophages and human THP-1 cell-derived macrophages with a toll-like receptor 4 (TLR4) antagonist attenuated pro-inflammatory cytokine induction and the activation of intracellular signaling by S1 and lipopolysaccharide. Similar results were obtained in experiments using TLR4 siRNA-transfected murine RAW264.7 macrophages. In contrast, TLR2 neutralizing antibodies could not abrogate the S1-induced pro-inflammatory cytokine induction in either RAW264.7 or THP-1 cell-derived macrophages. These results suggest that SARS-CoV-2 spike protein S1 subunit activates TLR4 signaling to induce pro-inflammatory responses in murine and human macrophages. Therefore, TLR4 signaling in macrophages may be a potential target for regulating excessive inflammation in COVID-19 patients.

## 1 Introduction

Coronavirus disease 2019 (COVID-19), which has now spread globally, is an infectious disease caused by a novel type of coronavirus referred as severe acute respiratory syndrome coronavirus 2 (SARS-CoV-2). The most frequent symptom of severe COVID-19 is pneumonia, accompanied by fever, cough, and dyspnea [1]. Although the majority of infected patients either do not experience pneumonia or exhibit mild symptoms, a portion of patients develop critical complications, such as respiratory failure, systemic shock, or multiple organ failure [2]. Growing evidence suggests that excessive host pro-inflammatory responses cause disease severity and mortality in patients [1, 3].

Compared with moderate cases, severe cases show markedly higher circulating levels of tumor necrosis factor-α (TNF-α), interleukin-6 (IL-6), IL-10, and the soluble form of IL-2 receptor [4], exhibiting features similar to those of cytokine storm syndromes, such as macrophage activation syndrome [1]. Indeed, critically ill patients are characterized by a depletion of tissue-resident alveolar macrophages and a remarkably increased proportion of recruited pro-inflammatory monocyte-derived macrophages in the bronchoalveolar lavage fluid [5]. Therefore, it is estimated that a decline in clearance of dead lung cells and subsequent diffusion of pro-inflammatory lipid mediators from these cells mediate the dysregulated recruitment and pro-inflammatory activation of monocyte-derived macrophages [6].

On the other hand, the propagated virus itself can also provoke pro-inflammatory responses in macrophages, as macrophages produce pro-inflammatory cytokines by detecting a broad range of pathogens using pattern recognition receptors, such as the toll-like receptors (TLRs). SARS-CoV-2 entry into host cells depends on binding of the surface subunit S1 of the spike protein to its receptor, human angiotensin-converting enzyme 2 (ACE2) [7], as in the case of SARS-CoV entry [8]. Previous studies have demonstrated that SARS-CoV spike recombinant protein induces macrophage pro-inflammatory responses by activating the nuclear factor-κB (NF-κB) signaling pathway [9, 10]. Dosch et al. [9] also reported that SARS-CoV spike protein induced IL-8 production in human embryonic kidney 293 cells transfected with the human TLR2 gene. Moreover, a recent in silico analysis suggested that the SARS-CoV-2 spike protein has the potential to interact with certain members of human TLRs, including TLR4, in addition to ACE2 [11].

However, it remains to be elucidated whether SARS-CoV-2 spike protein S1 subunit (hereafter S1) induces pro-inflammatory responses in macrophages, and which TLRs and signaling pathways mediate these responses. In the present study, we found that SARS-CoV-2 S1 protein strongly provokes the production of pro-inflammatory mediators and the activation of NF-κB and stress-activated mitogen-activated protein kinase (MAPK) signaling pathways via TLR4, in a manner similar to lipopolysaccharide (LPS)-mediated induction of inflammatory responses. Our findings support the idea that TLR4 signaling in macrophages may be a potential target for regulating excessive inflammation during SARS-CoV-2 infection.

## 2 Materials and methods

### 2.1 Animal care and use

Adult (8–12-week-old) male C57BL/6J mice (Sankyo Labo Service, Tokyo, Japan) were housed at a temperature of 23–25 °C and a humidity of 50–60% with a fixed light/dark cycle (light, 7:00–19:00; dark, 19:00–7:00). Food and water were available ad libitum. This study was approved by the Experimental Animal Ethics Committee in Kyorin University (No. 189, 2020). All experiments described below were carried out in accordance with the Guiding Principles for the Care and Use of Animals approved by the Council of the Physiological Society of Japan, based on the Declaration of Helsinki, 1964.

### 2.2 Preparation and culture of peritoneal exudate macrophages

Two milliliters of 4.05% autoclaved Thioglycollate Medium Brewer Modified (Becton, Dickinson and Company, Franklin Lakes, NJ, USA) was injected intraperitoneally into the mice, and the mice were housed for four days [12, 13]. After the mice were euthanized by cervical dislocation, peritoneal exudate cells were harvested by sterile lavage of the peritoneal cavity with ice-cold Dulbecco's modified Eagle's medium (DMEM; Nacalai Tesque, Kyoto, Japan). The cells were washed once with ice-cold DMEM, resuspended in DMEM supplemented with 1% heat-inactivated fetal bovine serum (FBS; BioWest, Nuaillé, France), 100 units/ml penicillin (Nacalai Tesque), and 100 μg/ml streptomycin (Nacalai Tesque), and cultured at 37 °C in a humidified incubator containing 5% CO_2_ for 1 h. After the nonadherent cells were removed, peritoneal exudate macrophages were used in experiments.

### 2.3 Cell lines and culture

The human monocytic cell line THP-1 cells (Japanese Collection of Research Bioresources Cell Bank, Osaka, Japan) were differentiated into macrophages by culturing with 5 ng/ml of phorbol 12-myristate 13-acetate (PMA; FUJIFILM Wako Pure Chemical, Osaka, Japan) in DMEM supplemented with 10% heat-inactivated FBS and antibiotics for 72 h. After the residual PMA was removed, THP-1 cell-derived macrophages were used in experiments. The murine macrophage cell line RAW264.7 cells (American Type Culture Collection, Manassas, VA, USA) were maintained in DMEM supplemented with 10% heat-inactivated FBS and antibiotics.

### 2.4 Agents and treatment

Murine peritoneal exudate macrophages or human THP-1 cell-derived macrophages were stimulated with 0.1–1 μg/ml of SARS-CoV-2 spike recombinant protein S1 subunit (Arigo Biolaboratories, Hsinchu City, Taiwan), 100 ng/ml of a TLR4 agonist LPS from *Escherichia coli* 055:B5 (LPS-B5 Ultrapure; InvivoGen, San Diego, CA, USA), or 10 ng/ml of a TLR2 agonist Pam2CSK4 (InvivoGen) to induce pro-inflammatory responses. Cells were treated with 2 μM BAY 11-7082 (Abcam, Cambridge, UK), 10 μM SP600125 (Sigma-Aldrich), 0.1 or 1 μg/ml of LPS from *Rhodobacter sphaeroides* (LPS-RS Ultrapure; InvivoGen), or 5 μg/ml of anti-murine or anti-human TLR2 neutralizing antibodies (InvivoGen) to block NF-κB, c-Jun N-terminal kinase (JNK), TLR4, or TLR2 signaling, respectively. The final concentrations of vehicles (H_2_O or dimethyl sulfoxide) to dissolve these agents were equivalent (less than 0.1%) in the culture medium among experimental groups.

### 2.5 Enzyme-linked immunosorbent assay (ELISA)

Cell culture supernatants were collected after centrifuging at 300 × *g* for 20 min. Concentrations of TNF-α, IL-6, and IL-1β were measured using the Quantikine Mouse TNF-α ELISA Kit (R&D Systems, Minneapolis, MN, USA), Quantikine Mouse IL-6 ELISA Kit (R&D Systems), and Quantikine Mouse IL-1β ELISA Kit (R&D Systems), respectively [13]. Since the detection limits of TNF-α, IL-6, and IL-1β using ELISA are 10.9–700 pg/ml, 7.8–500 pg/ml, and 7.8–500 pg/ml, respectively, the supernatants were diluted 50 times before TNF-α and IL-6 concentrations were measured.

### 2.6 Griess test

Nitrite concentrations in cell culture supernatants were determined using Griess-Romijn Nitrite Reagent (FUJIFILM Wako Pure Chemical) with sodium nitrite as a standard [14].

### 2.7 Reverse transcription and real-time polymerase chain reaction (PCR)

Total cellular RNA was extracted using RNAiso Plus reagent (TaKaRa Bio, Shiga, Japan). One microgram of total cellular RNA was converted to single-stranded cDNA using the PrimeScript 1st Strand cDNA Synthesis Kit (Takara Bio). cDNA (1 μl) was amplified using the FastStart Universal Probe Master (Roche Life Science, Indianapolis, IN, USA) in the 7500 Real Time PCR System (Thermo Fisher Scientific, Waltham, MA, USA). The PCR incubations were as follows: 50 °C for 2 min and 95 °C for 15 s, followed by 45 cycles of 95 °C for 15 s and 60 °C for 1 min. The fluorescent probes and primers are listed in Supplementary Table 1. The mRNA expression levels of target genes were calculated as the ratio of their values to that of 18S rRNA as an internal control.

### 2.8 Preparation of nuclear and cytosolic proteins

Nuclear and cytosolic proteins were prepared as previously described [12, 15]. Cytosolic proteins were extracted in lysis buffer containing 10 mM HEPES–KOH (pH 7.8), 10 mM KCl, 2 mM MgCl_2_, 0.1 mM ethylenediaminetetraacetic acid (EDTA), and 0.1% Nonidet P-40 supplemented with a protease inhibitor cocktail (Nacalai Tesque). After low-speed centrifugation (200 × *g*) at 4 °C for 5 min, sediments containing nuclei were resuspended in wash buffer containing 250 mM sucrose, 10 mM HEPES–KOH (pH 7.8), 10 mM KCl, 2 mM MgCl_2_, and 0.1 mM EDTA supplemented with inhibitors. After low-speed centrifugation (200 ×*g*) at 4 °C for 5 min, the sediments were resuspended in nuclear extraction buffer containing 50 mM HEPES–KOH (pH 7.8), 420 mM KCl, 5 mM MgCl_2_, 0.1 mM EDTA, and 20% glycerol supplemented with inhibitors, and rotated at 4 °C for 30 min. After high-speed centrifugation (13,000 × *g*) at 4 °C for 15 min, the supernatants were used as nuclear proteins. The concentrations of nuclear and cytosolic proteins were determined using a BCA protein assay kit (Pierce Biotechnology, Rockford, IL, USA).

### 2.9 Western blotting

Whole cellular, nuclear, or cytosolic proteins (10 μg) were separated by electrophoresis on a sodium dodecyl sulfate-polyacrylamide gel and then transferred onto a polyvinylidene difluoride membrane (GE Healthcare, Little Chalfont, UK). After blocking with 5% bovine serum albumin, each membrane was reacted with primary antibodies for 1 h (Supplementary Table 2). Secondary antibodies conjugated with horseradish peroxidase (Jackson ImmunoResearch Laboratories, West Grove, PA, USA) were applied at a 1:20,000 dilution for 30 min. The membrane was incubated with Clarity Western ECL Substrate (Bio-Rad Laboratories, Hercules, CA, USA) and imaged using LuminoGraph I (ATTO, Tokyo, Japan). The density of each protein band was quantified using ImageJ software (National Institutes of Health, Bethesda, MD, USA). The expression or phosphorylation levels of target proteins were calculated as the ratio of their values to those of glyceraldehyde-3-phosphate dehydrogenase (GAPDH) or the corresponding total protein as a loading control, respectively. Yin Yang 1 (YY1) and GAPDH were used as loading controls for nuclear and cytosolic proteins, respectively.

### 2.10 Fluorescence immunomicroscopy

The cells were fixed with 4% paraformaldehyde for 15 min and then permeabilized with methanol at -20 °C for 10 min. After blocking with 1% bovine serum albumin, primary antibody against p65 (8242S; Cell Signaling Technology, Danvers, MA, USA) was applied at a 1:400 dilution for 1 h. Secondary antibody conjugated with Alexa Fluor 568 (Abcam) was applied at a 1:1,000 dilution with 3 μM Nuclear Green DCS1 (Abcam) for 30 min. After mounting, subcellular localization of p65 and nucleus was visualized using a Nikon BioStation IM (NIKON, Tokyo, Japan) with FL2 (orange-red) and FL1 (green) detectors, respectively [15].

### 2.11 RNA interference (RNAi)

The pre-designed Stealth RNAi siRNA (Thermo Fisher Scientific) or Stealth RNAi negative control duplex (Thermo Fisher Scientific) were transfected into RAW264.7 cells by a reverse transfection protocol using HiPerFect Transfection Reagent (Qiagen, Hilden, Germany) with a final concentration of 10 nM, in accordance with the manufacturer's instructions. The sequences of murine TLR4 siRNA duplex were 5′-CCU AGU ACA UGU GGA UCU UUC UUA U-3′ (sense) and 5′-AUA AGA AAG AUC CAC AUG UAC UAG G-3′ (antisense).

### 2.12 Statistical analysis

Experimental data are presented as the mean ± standard error of the mean (SEM). Differences between two groups were assessed using the Student's *t*-test. Comparisons among at least three groups were tested by one-way analysis of variance (ANOVA), and then post hoc comparisons to determine significant differences between several experimental groups and the control group and between two groups were performed using Dunnett's test and Bonferroni test, respectively. Differences with *p*-values less than 0.05 were considered statistically significant.

## 3 Results

### 3.1 SARS-CoV-2 spike protein S1 subunit induces production of pro-inflammatory mediators in murine macrophages

To assess the purity of SARS-CoV-2 spike recombinant protein S1 subunit, we electrophoresed 1 μg of the protein after denaturation, and confirmed that a single band was detected at approximately 75 kDa (Supplemental Fig. 1A). In addition, since endotoxin levels of the recombinant protein were less than 0.1 EU/μg protein, we confirmed that 10 pg/ml of LPS, which might be present due to contamination, does not cause TNF-α mRNA induction in murine peritoneal exudate macrophages (Supplemental Fig. 1B).

**Figure 1:**
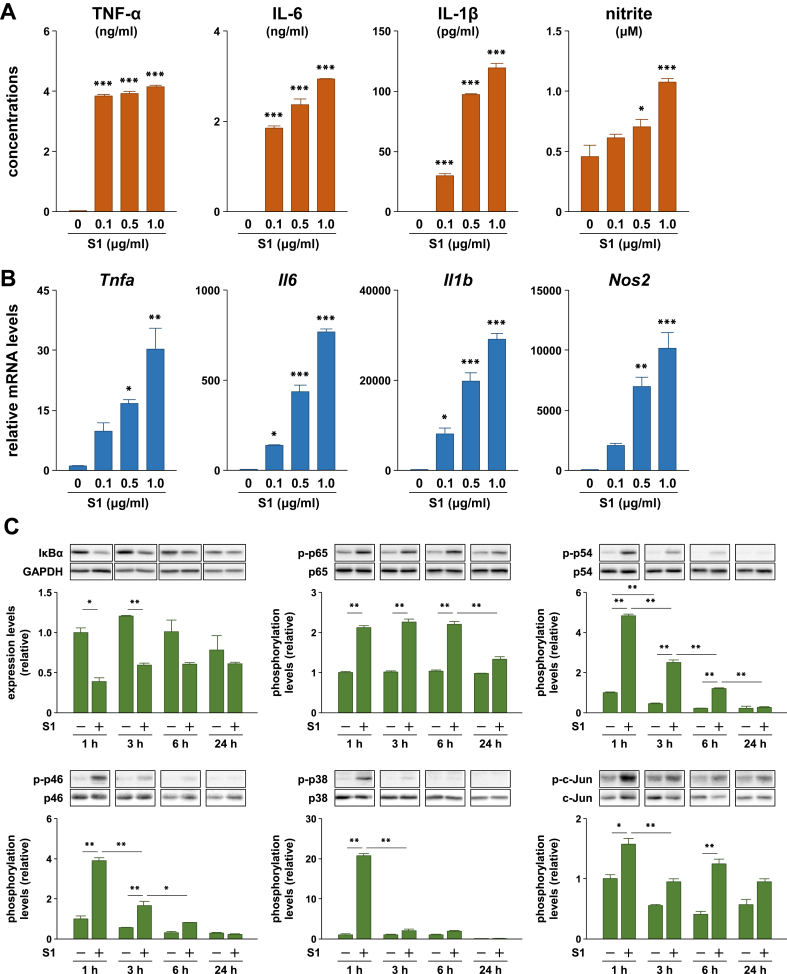
Effects of SARS-CoV-2 spike protein S1 subunit on pro-inflammatory responses in murine peritoneal exudate macrophages. (A) Pro-inflammatory cytokine and nitrite levels in cell culture supernatants and (B) transcript levels of target genes following stimulation of cells with 0, 0.1, 0.5, or 1.0 μg/ml of S1 for 24 h. (C) Expression and phosphorylation levels of target proteins in cells stimulated with 100 ng/ml of S1 for 1, 3, 6, or 24 h. Data are shown as the mean ± SEM (*n* = 3). ∗*p* < 0.05, ∗∗*p* < 0.01, ∗∗∗*p* < 0.001, by one-way ANOVA and Dunnett's test (A and B) or Bonferroni test (C).

We analyzed the effects of S1 on production of pro-inflammatory mediators, including TNF-α, IL-6, IL-1β, and nitric oxide, in murine peritoneal exudate macrophages. Upon stimulation with different concentrations (0, 0.1, 0.5, or 1.0 μg/ml) of S1 for 24 h, pro-inflammatory protein levels in the cell culture supernatants were significantly elevated, compared with those in controls in a dose-dependent manner (Figure 1A). Transcript levels of the *Tnfa*, *Il6*, *Il1b*, and *Nos2* also showed similar increases (Figure 1B).

### 3.2 SARS-CoV-2 spike protein S1 subunit activates NF-κB and stress-activated MAPK signaling pathways in murine macrophages

**Figure 2:**
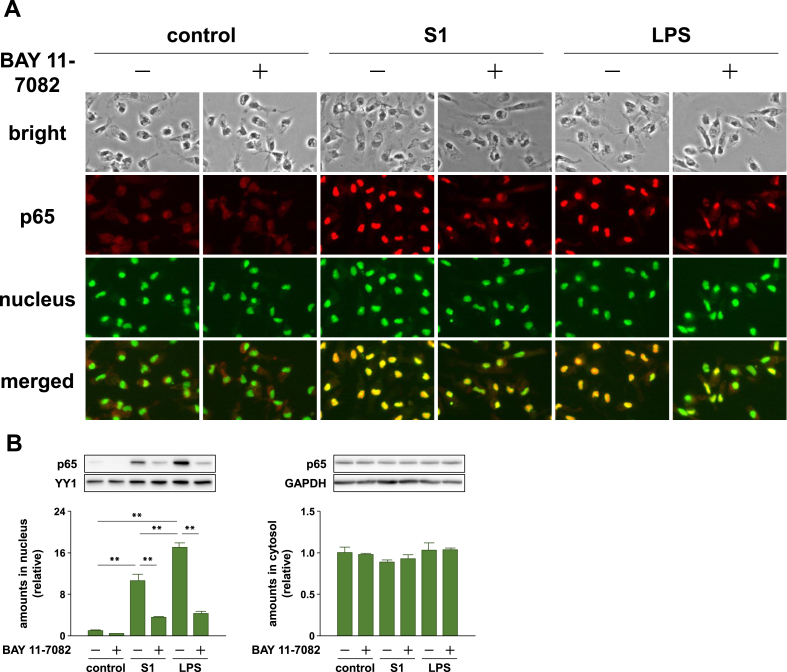
Effects of an inhibitor of NF-κB (BAY 11-7082) on SARS-CoV-2 spike protein S1 subunit-induced nuclear import of p65 in murine peritoneal exudate macrophages and RAW264.7 cells. (A) Murine peritoneal exudate macrophages were pretreated with 2 μM BAY 11-7082 for 30 min, and then stimulated with 100 ng/ml of S1 or LPS for 1 h. Subcellular localization of p65 in cells was visualized by fluorescence immunomicroscopy. (B) RAW264.7 cells were treated with 10 μM BAY 11-7082 for 30 min, and then stimulated with 100 ng/ml of S1 or LPS for 1 h in the presence of the inhibitor. Amount of p65 in the nucleus and cytosol was analyzed by western blotting. Data are shown as the mean ± SEM (*n* = 4). ∗∗*p* < 0.01, by one-way ANOVA and Bonferroni test.

We next analyzed the effects of S1 on NF-κB and stress-activated MAPK signaling pathways, which regulate the expression of pro-inflammatory mediators. Stimulation of murine peritoneal exudate macrophages with 100 ng/ml of S1 induced IκBα degradation and an increase in p65 phosphorylation 1–6 h after stimulation (Figure 1C). In addition, as detected by fluorescence immunomicroscopy, p65 translocated into the nucleus in almost all cells stimulated with S1 or LPS for 1 h (Figure 2A). Western blotting also revealed nuclear accumulation of p65 in murine RAW264.7 macrophages stimulated with S1 or LPS for 1 h (Figure 2B). Although both JNK and p38 exhibited a transient phosphorylation 1 h after stimulation, JNK phosphorylation gradually attenuated during the 6 h following stimulation (Figure 1C). Phosphorylation of the downstream transcription factor c-Jun showed a pattern of increase similar to that observed for JNK (Figure 1C).

### 3.3 SARS-CoV-2 spike protein S1 subunit induces production of pro-inflammatory cytokines via NF-κB and JNK pathways in murine macrophages

**Figure 3:**
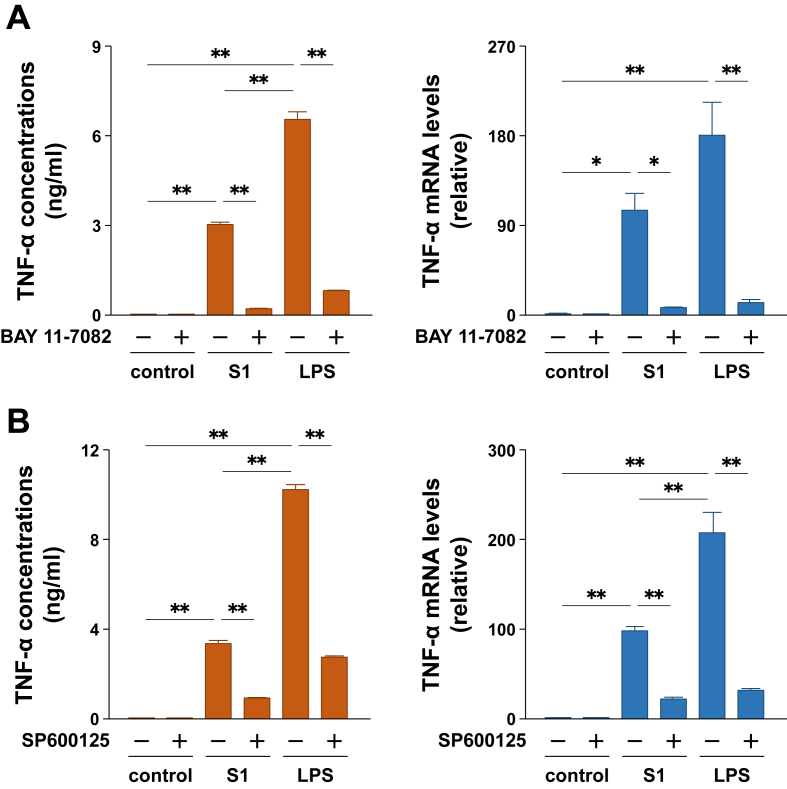
Effects of inhibitors of NF-κB (BAY 11-7082) and JNK (SP600125) on SARS-CoV-2 spike protein S1 subunit-induced production of TNF-α in murine peritoneal exudate macrophages. (A) Cells were pretreated with 2 μM BAY 11-7082 for 30 min, and then stimulated with 100 ng/ml of S1 or LPS for 6 h. (B) Cells were treated with 10 μM SP600125 for 1 h, and then stimulated with 100 ng/ml of S1 or LPS for 6 h in the presence of SP600125. TNF-α levels in cell culture supernatants were measured by ELISA. TNF-α mRNA levels were assessed by real-time PCR. Data are shown as the mean ± SEM (*n* = 3 or 4). ∗*p* < 0.05, ∗∗*p* < 0.01, by one-way ANOVA and Bonferroni test.

To determine whether the S1-induced production of pro-inflammatory mediators is regulated by the NF-κB and JNK signaling pathways, we analyzed the effects of inhibitors of NF-κB (BAY 11-7082) and JNK (SP600125). When murine peritoneal exudate macrophages were pretreated with 2 μM BAY 11-7082 for 30 min prior to stimulation with 100 ng/ml of S1 or LPS, the increases in TNF-α secretion and mRNA levels (Figure 3A), as well as the nuclear translocation of p65 (Figure 2A), were dramatically suppressed. The nuclear accumulation of p65 in murine RAW264.7 macrophages stimulated with S1 or LPS was also suppressed by BAY 11-7082 treatment (Figure 2B). Moreover, treatment of the cells with 10 μM SP600125 for 1 h completely inhibited JNK and c-Jun phosphorylation induced by 100 ng/ml of S1 or LPS treatment (Supplemental Fig. 2). Lack of JNK/c-Jun phosphorylation was accompanied by a dramatic suppression of the increases in TNF-α secretion and mRNA levels after 6 h of stimulation (Figure 3B).

### 3.4 SARS-CoV-2 spike protein S1 subunit induces pro-inflammatory responses via TLR4 signaling in murine and human macrophages

**Figure 4:**
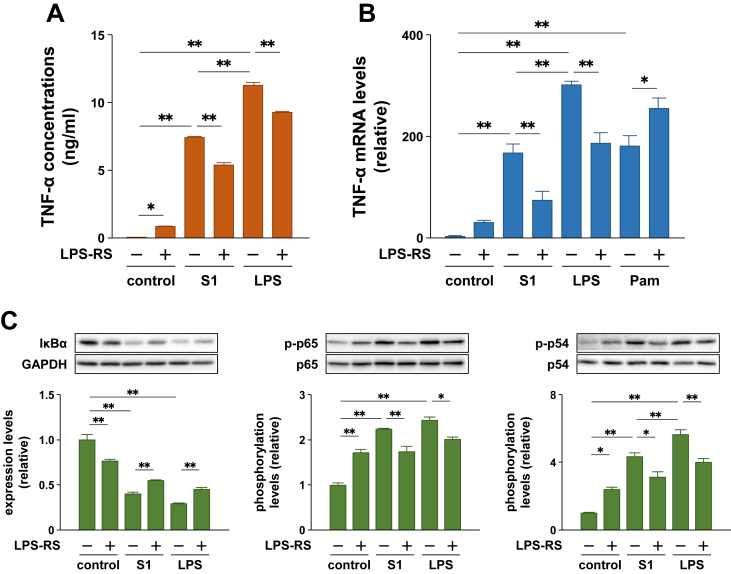
Effects of a TLR4 antagonist LPS-RS on SARS-CoV-2 spike protein S1 subunit-induced pro-inflammatory responses in murine peritoneal exudate macrophages. Cells were pretreated with 100 ng/ml of LPS-RS for 1 h, and then stimulated with 100 ng/ml of S1 or LPS for 6 h (A and B) or 1 h (C). Concentration of Pam2CSK4 is 10 ng/ml (B). (A) TNF-α levels in cell culture supernatants were measured by ELISA. (B) TNF-α mRNA levels were analyzed by real-time PCR. (C) Expression and phosphorylation levels of target proteins were analyzed by western blotting. Data are shown as the mean ± SEM (*n* = 3 or 4). ∗*p* < 0.05, ∗∗*p* < 0.01, by one-way ANOVA and Bonferroni test.

To examine whether the S1-induced pro-inflammatory responses in macrophages are mediated by TLR4 signaling, as is the case with LPS, we analyzed the effects of a TLR4 antagonist, LPS-RS. Although LPS-RS itself moderately induced the secretion and mRNA expression of TNF-α and activated the NF-κB and JNK signaling in murine peritoneal exudate macrophages, equivalent concentrations (100 ng/ml) of LPS-RS significantly suppressed the pro-inflammatory responses stimulated by S1 and LPS, but not by a TLR2 agonist Pam2CSK4 (Figure 4A–C).

**Figure 5:**
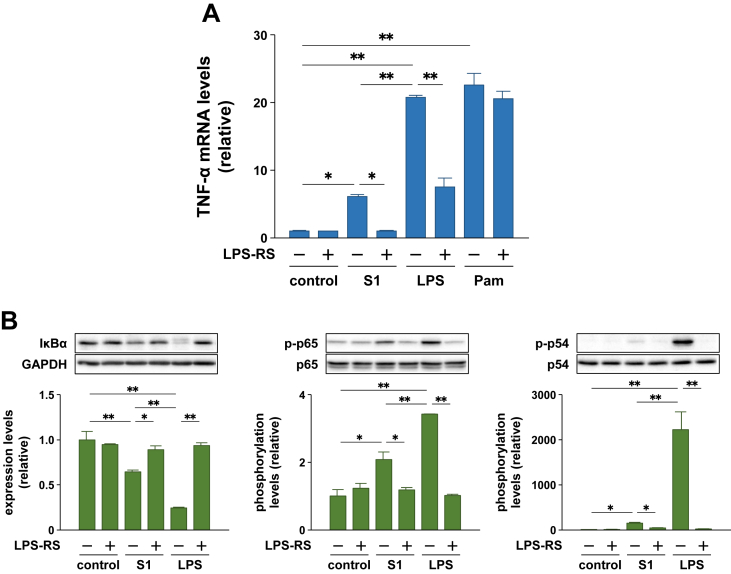
Effects of a TLR4 antagonist LPS-RS on SARS-CoV-2 spike protein S1 subunit-induced pro-inflammatory responses in human THP-1 cell-derived macrophages. Cells were pretreated with 1 μg/ml of LPS-RS for 30 min, and then stimulated with 100 ng/ml of S1 or LPS for 6 h (A) or 1 h (B). Concentration of Pam2CSK4 is 10 ng/ml (A). (A) TNF-α mRNA levels were analyzed by real-time PCR. (B) Expression and phosphorylation levels of target proteins were analyzed by western blotting. Data are shown as the mean ± SEM (*n* = 3 or 4). ∗*p* < 0.05, ∗∗*p* < 0.01, by one-way ANOVA and Bonferroni test.

Human THP-1 cell-derived macrophages also responded to 100 ng/ml of S1 and induced TNF-α transcription after 6 h of stimulation, which was completely suppressed by pretreatment with 1 μg/ml of LPS-RS (Figure 5A). However, unlike LPS, Pam2CSK4-induced expression of TNF-α mRNA was not influenced by LPS-RS (Figure 5A). IκBα degradation and phosphorylation of p65 and JNK p54 subunit stimulated by S1 and LPS treatments were also markedly suppressed by LPS-RS (Figure 5B).

### 3.5 RNAi also reveals contribution of TLR4 signaling in SARS-CoV-2 spike protein S1 subunit-induced pro-inflammatory responses in murine macrophages

**Figure 6:**
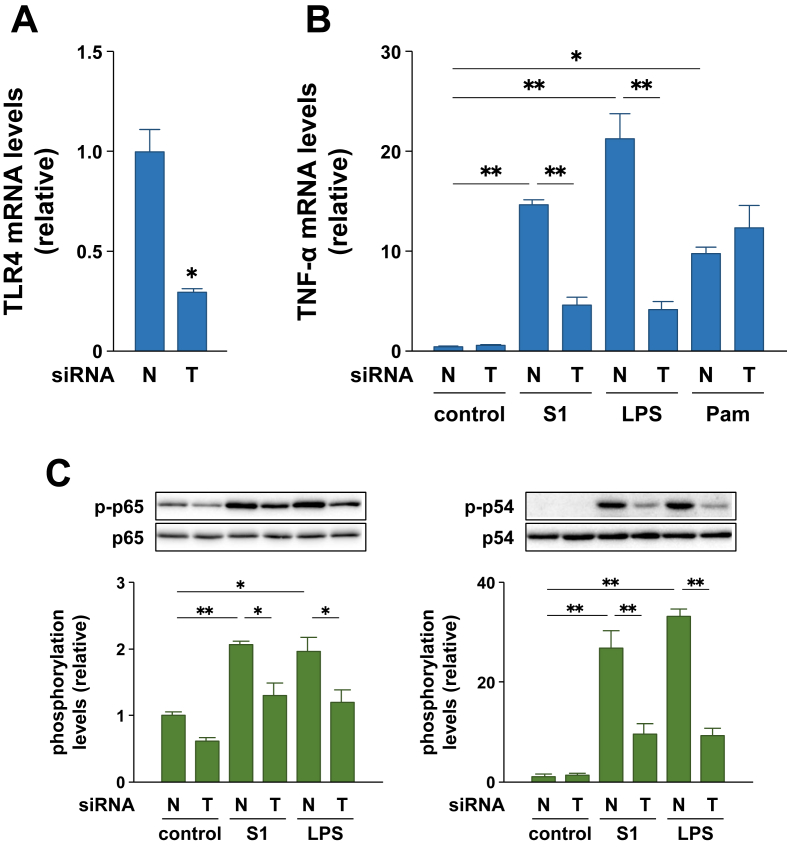
Effects of RNAi targeting TLR4 on SARS-CoV-2 spike protein S1 subunit-induced pro-inflammatory responses in RAW264.7 cells. Cells were transfected with 10 nM negative control (N) or TLR4 (T) siRNA for 48 h (A), and then stimulated with 100 ng/ml of S1 or LPS for 3 h (B) or 1 h (C). Concentration of Pam2CSK4 is 10 ng/ml (B). TLR4 (A) and TNF-α (B) mRNA levels were analyzed by real-time PCR. (C) Phosphorylation levels of target proteins were analyzed by western blotting. Data are shown as the mean ± SEM (*n* = 3). ∗*p* < 0.05, ∗∗*p* < 0.01, by Student's *t*-test (A) or one-way ANOVA and Bonferroni test (B and C).

To further confirm whether TLR4 signaling mediates the S1-indueced pro-inflammatory responses in macrophages, the effects of RNAi targeting TLR4 were investigated in murine RAW264.7 macrophages. RAW264.7 cells transfected with TLR4 siRNA showed significantly reduced *Tlr4* transcript levels (by 70%) compared to cells transfected with negative control siRNA (Figure 6A). When these cells were stimulated with 100 ng/ml of S1 or LPS for 3 h, *Tnfa* transcription in TLR4 siRNA-transfected cells was significantly lower, by 68% and 80% than that in negative control siRNA-transfected cells, respectively (Figure 6B). The expression levels of TNF-α mRNA after S1 and LPS stimulation were similar in TLR4 siRNA-transfected cells (Figure 6B). However, RNAi targeting TLR4 did not influence Pam2CSK4-induced expression of TNF-α mRNA (Figure 6B). Further, phosphorylation of p65 and JNK p54 subunit stimulated by S1 and LPS treatments was significantly suppressed to the same levels by RNAi targeting TLR4 (Figure 6C).

### 3.6 TLR2 does not play a major role in SARS-CoV-2 spike protein S1 subunit-induced pro-inflammatory responses in murine and human macrophages

**Figure 7:**
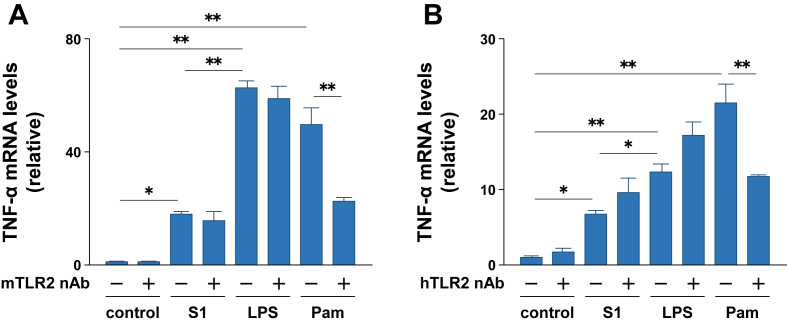
Effects of TLR2 neutralizing antibodies on SARS-CoV-2 spike protein S1 subunit-induced pro-inflammatory responses in murine RAW264.7 macrophages and human THP-1 cell-derived macrophages. Murine (A) and human (B) macrophages were pretreated with or without 5 μg/ml of anti-mouse and anti-human TLR2 neutralizing antibodies for 3 h, respectively, and then stimulated with 100 ng/ml of S1 or LPS for 3 h. Concentration of Pam2CSK4 is 10 ng/ml. TNF-α mRNA levels were analyzed by real-time PCR. Data are shown as the mean ± SEM (*n* = 4). ∗*p* < 0.05, ∗∗*p* < 0.01, by one-way ANOVA and Bonferroni test.

Since a previous study reported that SARS-CoV spike protein induced IL-8 production in the human embryonic kidney 293 cells transfected with the human TLR2 gene [9], we analyzed the effects of anti-TLR2 neutralizing antibodies on the S1-induced transcription of TNF-α mRNA in murine RAW264.7 macrophages and human THP-1 cell-derived macrophages. When RAW264.7 macrophages were treated with 5 μg/ml of anti-murine TLR2 neutralizing antibody for 3 h, TNF-α transcription induced by Pam2CSK4 stimulation was significantly suppressed (Figure 7A). However, S1- and LPS-induced expression of TNF-α mRNA was not abrogated by the TLR2 neutralizing antibody (Figure 7A). Similar results were obtained when THP-1 cell-derived macrophages were stimulated with S1, LPS, and Pam2CSK4 after treatment with an anti-human TLR2 neutralizing antibody (Figure 7B).

## 4 Discussion

This study demonstrated that SARS-CoV-2 spike protein S1 subunit induced production of pro-inflammatory mediators and activation of NF-κB and stress-activated MAPK signaling in murine primary macrophages. TNF-α production induced by S1 and LPS was remarkably suppressed by NF-κB and JNK inhibitors, suggesting that S1 induces pro-inflammatory responses through TLR signaling. LPS-RS inhibits TLR4 signaling by competing with pyrogenic LPS for binding to TLR4 coreceptor myeloid differentiation factor 2 [16]. It is not clear why LPS-RS itself induced pro-inflammatory responses in murine peritoneal exudate macrophages, since “LPS-RS Ultrapure” is not contaminated with lipoteichoic acids and actually does not activate human THP-1 cell-derived macrophages. Nevertheless, LPS-RS successfully suppressed both S1-induced and LPS-induced pro-inflammatory responses without affecting the action of Pam2CSK4 in both murine and human macrophages.

S1 can induce a number of pro-inflammatory cytokines, which in turn may activate the intracellular signaling in a paracrine manner. However, the incomplete inhibitory effects of LPS-RS may be mainly caused by the lack of its potency, since the concentration and treating time of LPS-RS could not be increased due to its side effects on murine peritoneal exudate macrophages as described above. Therefore, we performed the RNAi experiments to confirm the role of TLR4 signaling in the S1-induced pro-inflammatory responses in murine macrophages. Indeed, RNAi targeting TLR4 markedly suppressed both S1-induced and LPS-induced pro-inflammatory responses to the same levels in murine RAW264.7 macrophages, along with decreasing the TLR4 mRNA expression level. Since the action of Pam2CSK4 was not influenced in this experiment, the TLR4 expression was specifically suppressed. These results indicate that S1 activates TLR4 signaling to induce pro-inflammatory responses in macrophages.

In addition to these TLR4 signaling inhibition experiments, we also demonstrated that TLR2 neutralizing antibodies abrogated the action of Pam2CSK4 but did not affect S1- and LPS-induced expression of TNF-α mRNA in both murine and human macrophages. Dosch et al. [9] previously reported that SARS-CoV spike protein induced IL-8 production in human embryonic kidney 293 cells transfected with the human TLR2 gene. Therefore, the receptors for spike proteins may differ between SARS-CoV and SARS-CoV-2. Alternatively, the mechanism of action of spike proteins may differ between macrophages expressing various endogenous TLRs and non-immune cell lines in which only TLR2 is forcibly expressed.

Coronavirus spike proteins typically contain 23–30 sites of N-linked glycosylation, depending on the species [17]. A recent study demonstrated that SARS-CoV-2 spike protein S1 subunit expressed on human cells was attached with O-glycans, not only N-glycans [18]. Therefore, it is not surprising that SARS-CoV-2 spike protein S1 subunit induces pro-inflammatory responses in macrophages by activating TLR4 signaling, considering the growing list of TLR4-activating viral glycoproteins, such as respiratory syncytial virus fusion protein, the Ebola virus glycoprotein, the vesicular stomatitis virus glycoprotein, and the dengue virus nonstructural protein 1 [19].

However, the SARS-CoV-2 spike recombinant protein S1 subunit used in this study was not glycosylated, since *E. coli* lacks endogenous glycosylation machinery. Although many of the endogenous proteins identified as TLR4 ligands, such as heat shock proteins [20, 21], fibronectin [22], and high mobility group box 1 [23], have at least one or more Asn-X-Ser/Thr sequons that serve as acceptors for N-linked glycosylation, some of the proteins, such as β-defensin 2 [24] and amyloid A3 [25], do not have these sequons. A previous study also showed that SARS-CoV spike recombinant protein purified from *E. coli* induced TNF-α and IL-6 expression by activating NF-κB signaling pathway in RAW264.7 cells, even though TLR4 has not been identified as its receptor; moreover, the pro-inflammatory action was abolished by heat denaturation of the spike protein [10]. These findings suggest that the high-order structure may be more important for the interaction between spike proteins and TLRs than the presence of glycans.

It is possible that S1 binds to ACE2, which mediates pro-inflammatory responses in macrophages. However, inoculation with SARS-CoV-2 caused weight loss and virus replication in the lungs of transgenic mice expressing human ACE2 but did not cause those of wild-type mice [26], indicating that S1 has no or low affinity for murine ACE2. In contrast, this study revealed that S1 causes pro-inflammatory responses in murine as well as human macrophages. Therefore, ACE2 may not be the main contributor to S1-mediated pro-inflammatory responses in macrophages. In addition, it can be considered that wild-type mice are not suitable for the experimental model of SARS-CoV-2 infection but may be useful for that of SARS-CoV-2 spike protein-induced systemic inflammation.

When murine peritoneal exudate macrophages or human THP-1 cell-derived macrophages were stimulated with the same concentrations (100 ng/ml) of S1 or LPS, the degree of induction of TNF-α expression was 1.5–3 times higher in cells treated with LPS, compared with those treated with S1. Given that the molecular weights of the S1 and LPS monomers are approximately 75 kDa and 10 kDa, respectively, our results suggest that SARS-CoV-2 spike protein S1 subunit has the potential to induce stronger responses than pyrogenic LPS derived from *E. coli* when compared by the activity per a molecule, even though it is not glycosylated.

It was also suggested that the propagated SARS-CoV-2 virus itself can cause macrophage activation syndrome by forming a complex of the spike protein S1 subunit with TLR4 and its co-receptors. Several studies have shown that people with diabetes are highly susceptible to infectious diseases [27], and that poor glycemic control is associated with a higher risk of infection-related morbidity and mortality, especially in the elderly population [28]. Moreover, leukocytes of both type 1 and type 2 diabetic mice show reduced phagocytic activity [29]. Therefore, it is possible that the propagated SARS-CoV-2 virus, which has overwhelmed the first line of immune systems, partially contributes to excessive host systemic inflammation. Although no drugs to block TLR4 signaling have been approved, dexamethasone, an anti-inflammatory steroid, is now expected to be effective in treating critically ill patients with COVID-19 [30].

Since COVID-19 has now spread worldwide, it is an urgent task to elucidate the mechanism underlying the severity and mortality of the disease. In this study, we found that SARS-CoV-2 spike protein S1 subunit provokes pro-inflammatory responses in macrophages by activating TLR4 signaling, similar to the action of LPS. These results suggest that TLR4 signaling in macrophages may be a potential target for regulating excessive host inflammation in COVID-19 patients.

## Declarations

### Author contribution statement

Ken Shirato: Conceived and designed the experiments; Performed the experiments; Analyzed and interpreted the data; Contributed reagents, materials, analysis tools or data; Wrote the paper.

Takako Kizaki: Analyzed and interpreted the data; Wrote the paper.

### Funding statement

Ken Shirato was supported by the 10.13039/501100001700Ministry of Education, Culture, Sports, Science and Technology, Japan (Grant-in-Aid for Scientific Research (C) 18K10856), the 10.13039/501100008882Meiji Yasuda Life Foundation of Health and Welfare (36th Research Grant) and the 10.13039/100008731Nakatomi Foundation (32nd Research Grant). Takako Kizaki was supported by the 10.13039/501100001700Ministry of Education, Culture, Sports, Science and Technology, Japan (Grant-in-Aid for Scientific Research (B) 17H02160).

### Data availability statement

Data included in article/supplementary material/referenced in article.

### Declaration of interests statement

The authors declare no conflict of interest.

### Additional information

No additional information is available for this paper.

## Appendix A Supplementary data

Supplementary Material Supplementary Material

Original images of blots in Fig Original images of blots in Fig
