# Supplementary material for: SARS-CoV-2 spike protein S1 subunit induces pro-inflammatory responses via toll-like receptor 4 signaling in murine and human macrophages
Source: Heliyon. 2021 Feb 2;7(2):e06187. doi: 10.1016/j.heliyon.2021.e06187 (PMC7887388; doi:10.1016/j.heliyon.2021.e06187)
Supplement: Supplementary Material [file mmc1.pptx]

## Slide 1
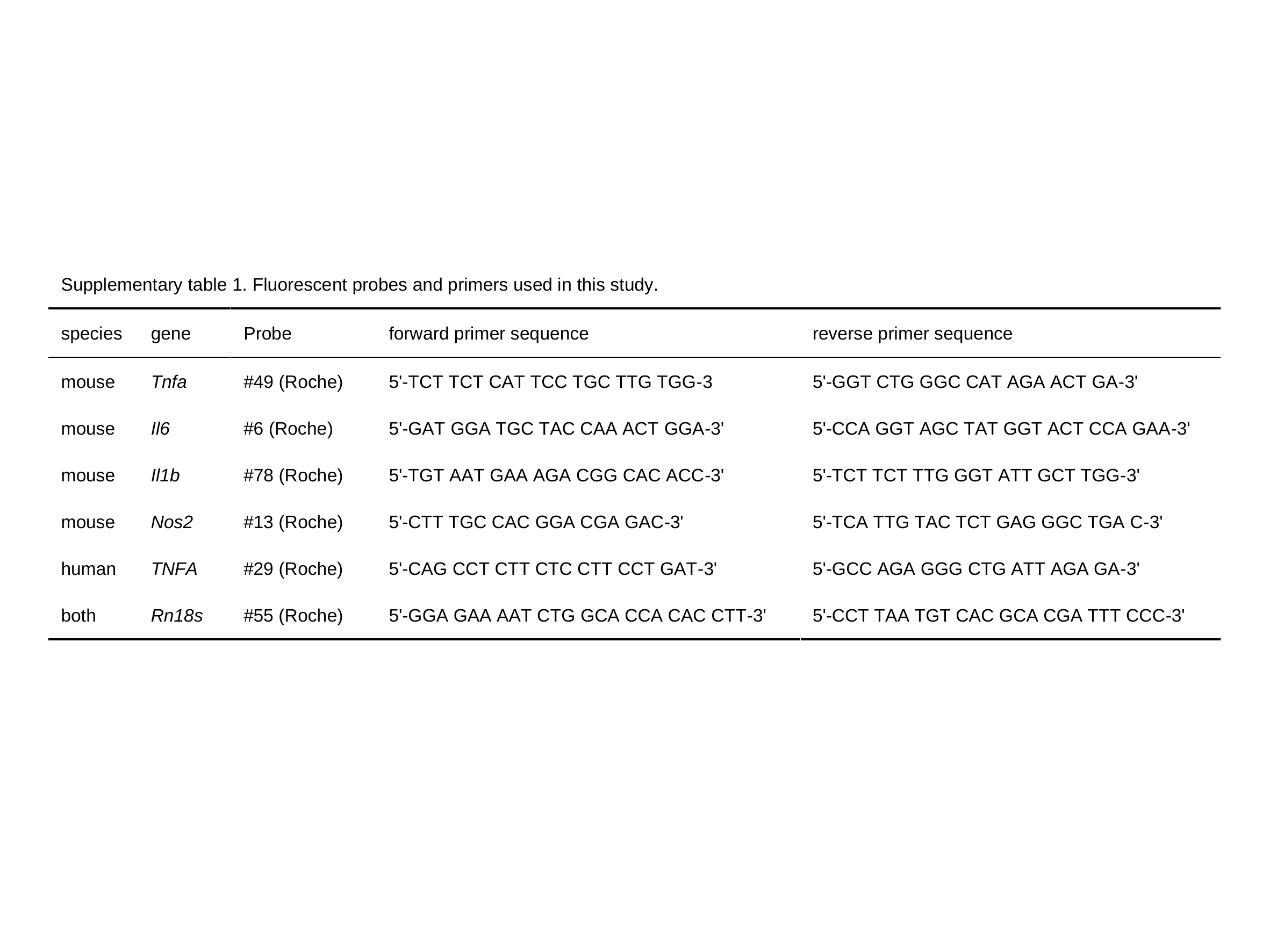

## Slide 2
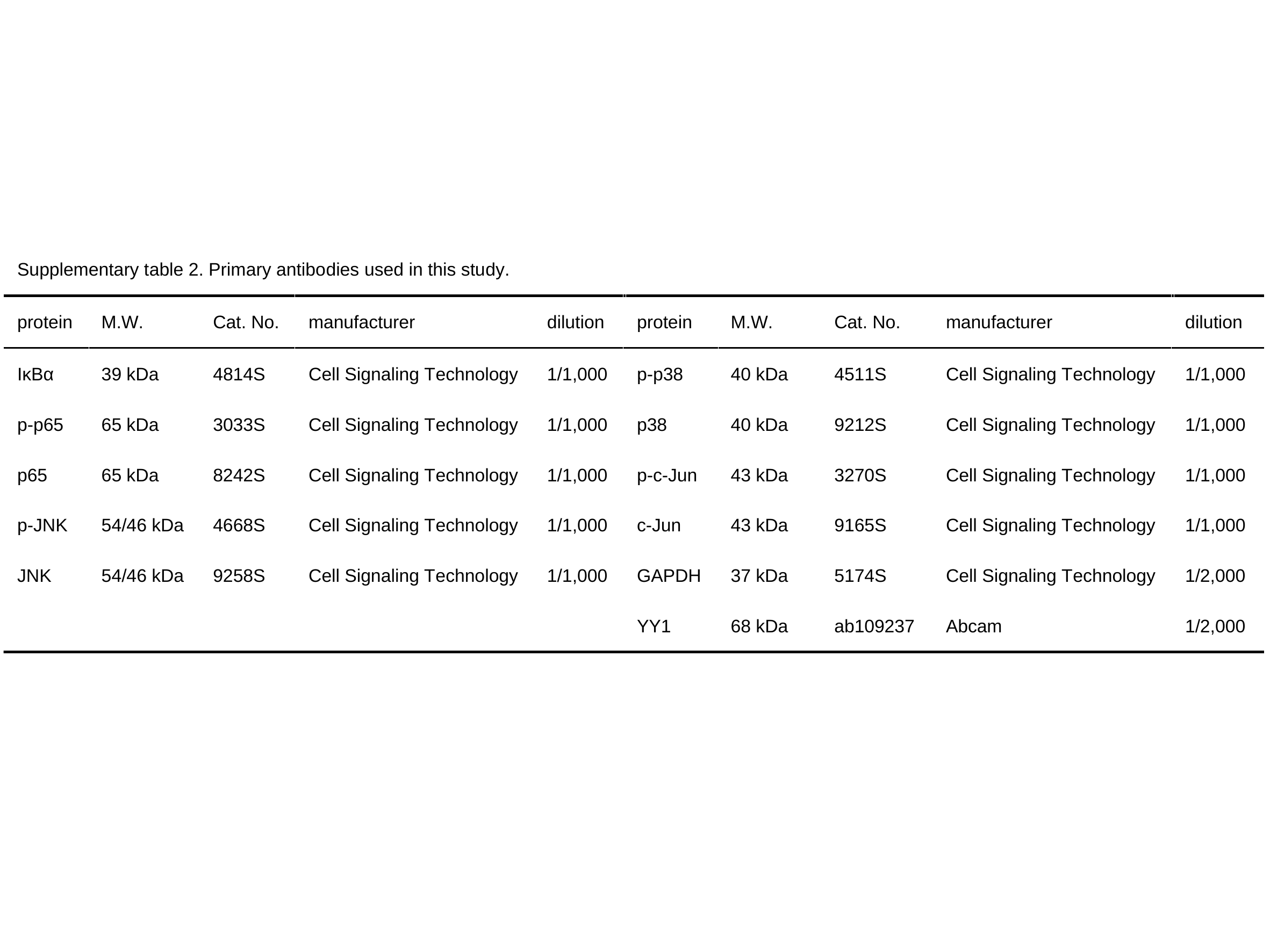

## Slide 3
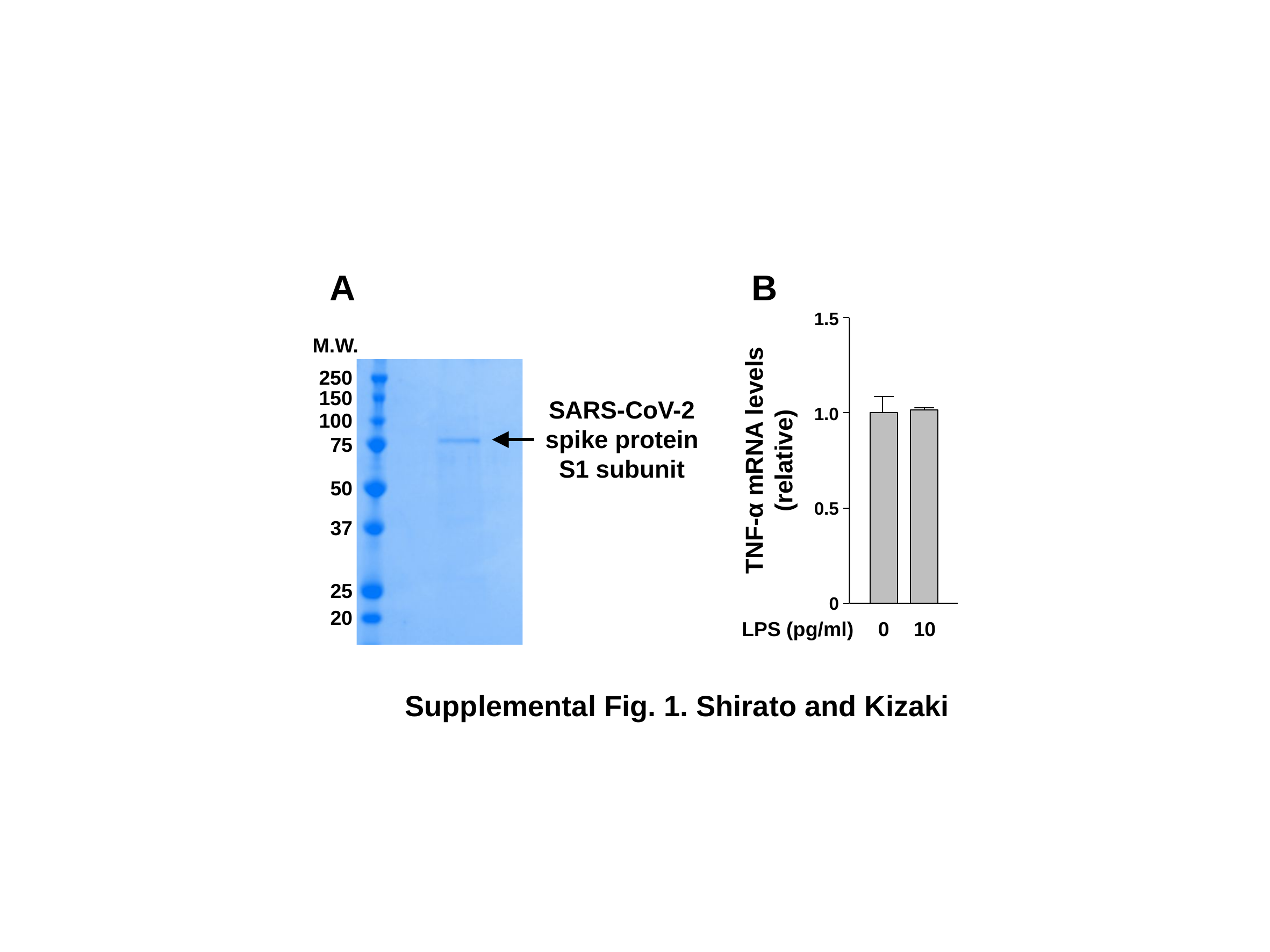

A
M.W.
250
150
SARS-CoV-2 spike protein S1 subunit
100
75
50
37
25
20
B
1.5
1.0
TNF-α mRNA levels (relative)
0.5
0
LPS (pg/ml)
0
10
Supplemental Fig. 1. Shirato and Kizaki

## Slide 4
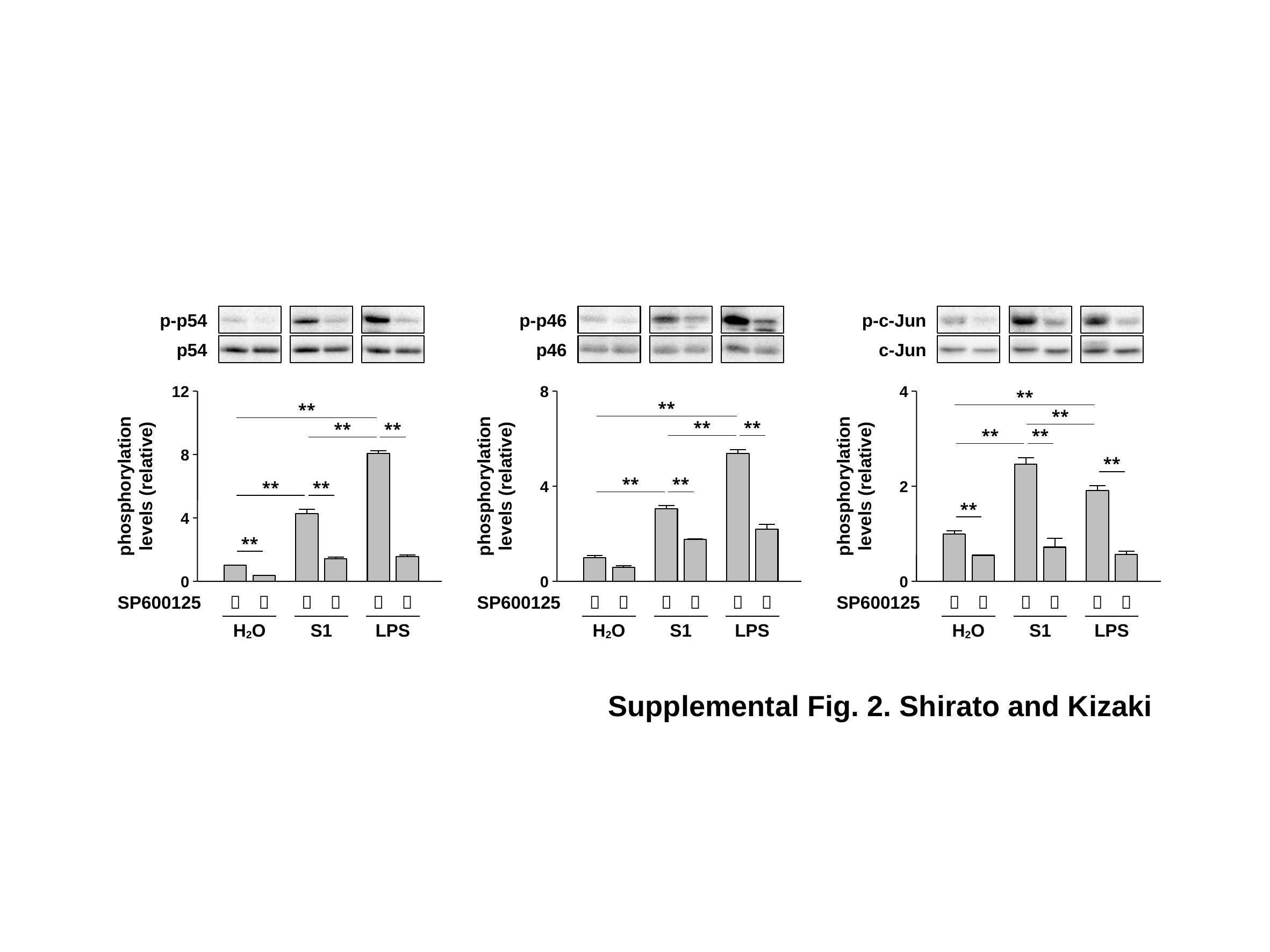

p-p54
p54
12
**
**
**
8
phosphorylation levels (relative)
**
**
4
**
0
SP600125
－
＋
－
＋
－
＋
H2O
S1
LPS
p-p46
p46
8
**
**
**
phosphorylation levels (relative)
**
**
4
0
SP600125
－
＋
－
＋
－
＋
H2O
S1
LPS
p-c-Jun
c-Jun
4
**
**
**
**
**
phosphorylation levels (relative)
2
**
0
SP600125
－
＋
－
＋
－
＋
H2O
S1
LPS
Supplemental Fig. 2. Shirato and Kizaki
