# Supplementary material for: SARS-CoV-2 spike protein S1 subunit induces pro-inflammatory responses via toll-like receptor 4 signaling in murine and human macrophages
Source: Heliyon. 2021 Feb 2;7(2):e06187. doi: 10.1016/j.heliyon.2021.e06187 (PMC7887388; doi:10.1016/j.heliyon.2021.e06187)
Supplement: Original images of blots in Fig [file mmc2.pptx]

## Slide 1
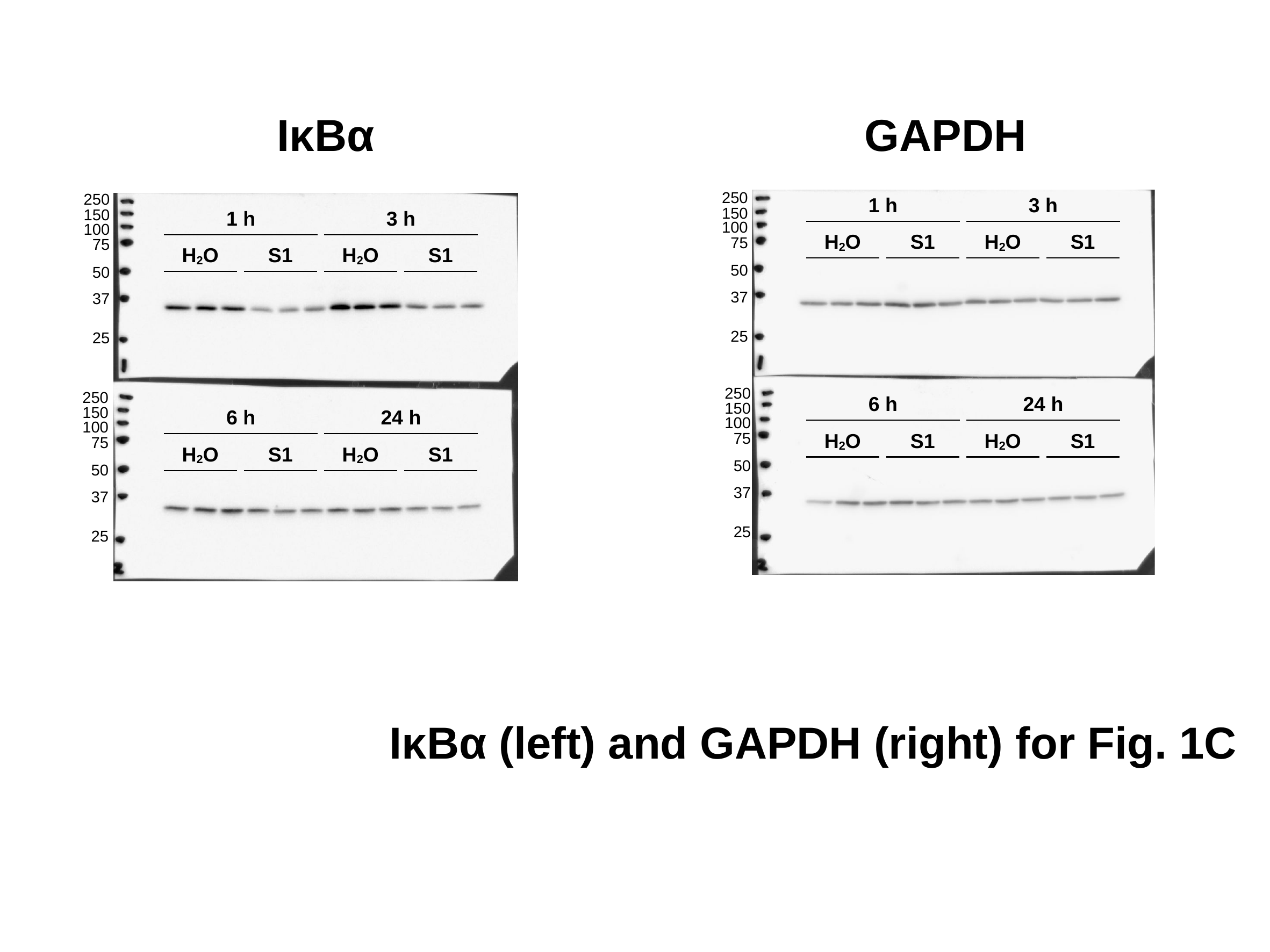

IκBα
GAPDH
250
250
1 h
3 h
H2O
S1
H2O
S1
150
150
1 h
3 h
H2O
S1
H2O
S1
100
100
75
75
50
50
37
37
25
25
250
250
6 h
24 h
H2O
S1
H2O
S1
150
150
6 h
24 h
H2O
S1
H2O
S1
100
100
75
75
50
50
37
37
25
25
IκBα (left) and GAPDH (right) for Fig. 1C

## Slide 2
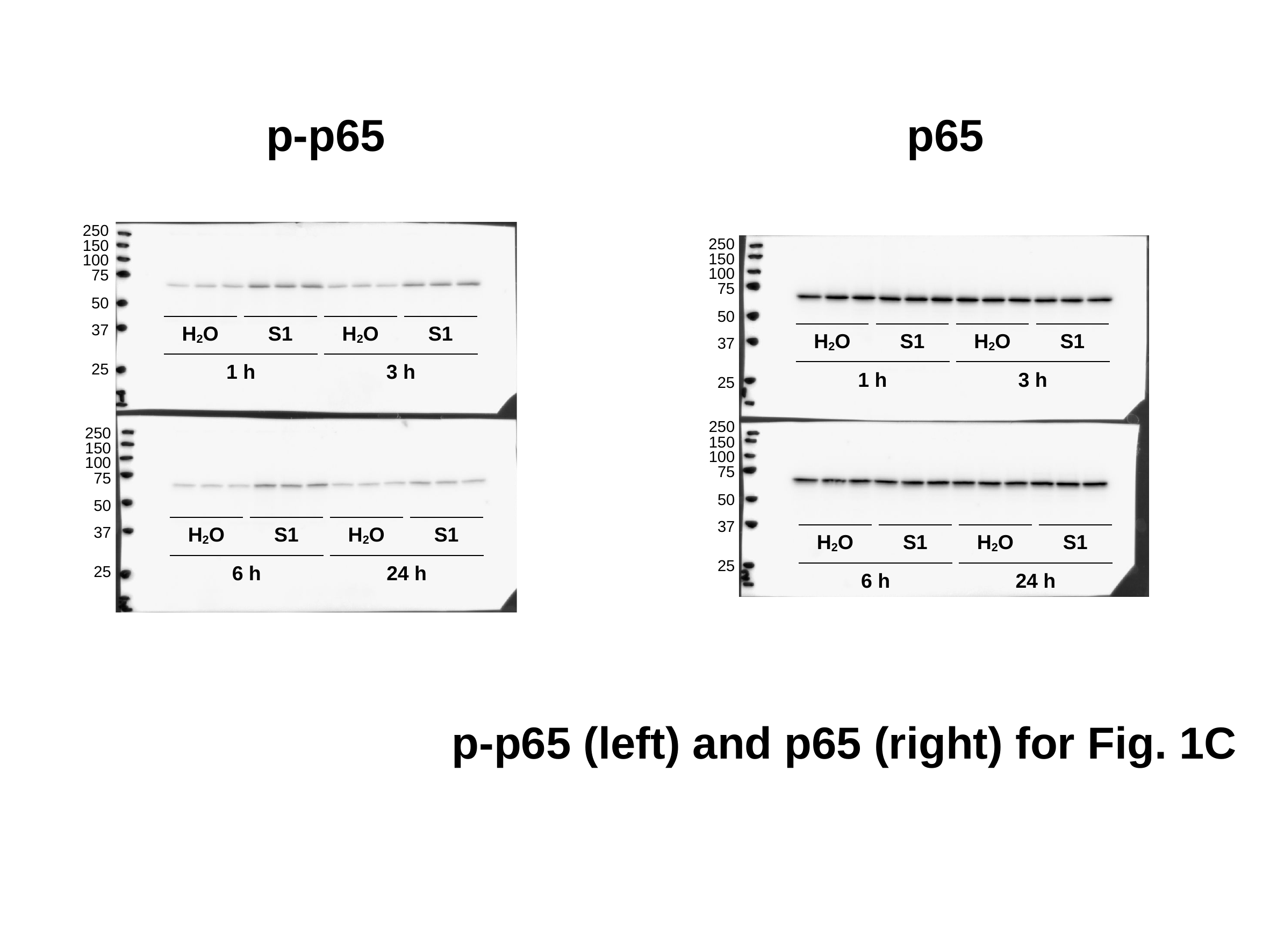

p-p65
p65
250
250
150
150
100
100
75
75
50
50
37
H2O
S1
H2O
S1
1 h
3 h
H2O
S1
H2O
S1
1 h
3 h
37
25
25
250
250
150
150
100
100
75
75
50
50
37
H2O
S1
H2O
S1
6 h
24 h
37
H2O
S1
H2O
S1
6 h
24 h
25
25
p-p65 (left) and p65 (right) for Fig. 1C

## Slide 3
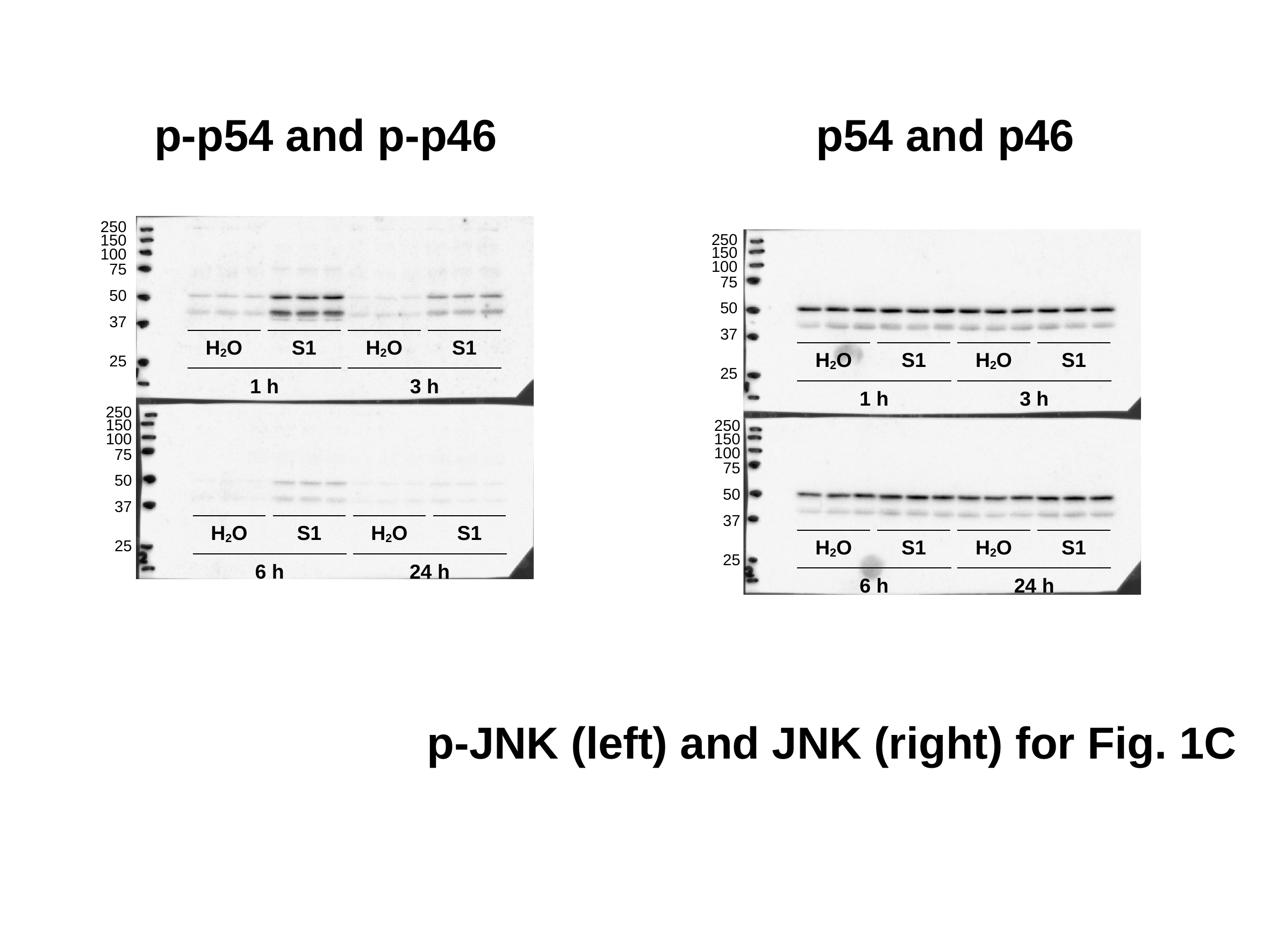

p-p54 and p-p46
p54 and p46
250
250
150
150
100
100
75
75
50
50
37
37
H2O
S1
H2O
S1
1 h
3 h
H2O
S1
H2O
S1
1 h
3 h
25
25
250
150
250
150
100
100
75
75
50
50
37
37
H2O
S1
H2O
S1
6 h
24 h
H2O
S1
H2O
S1
6 h
24 h
25
25
p-JNK (left) and JNK (right) for Fig. 1C

## Slide 4
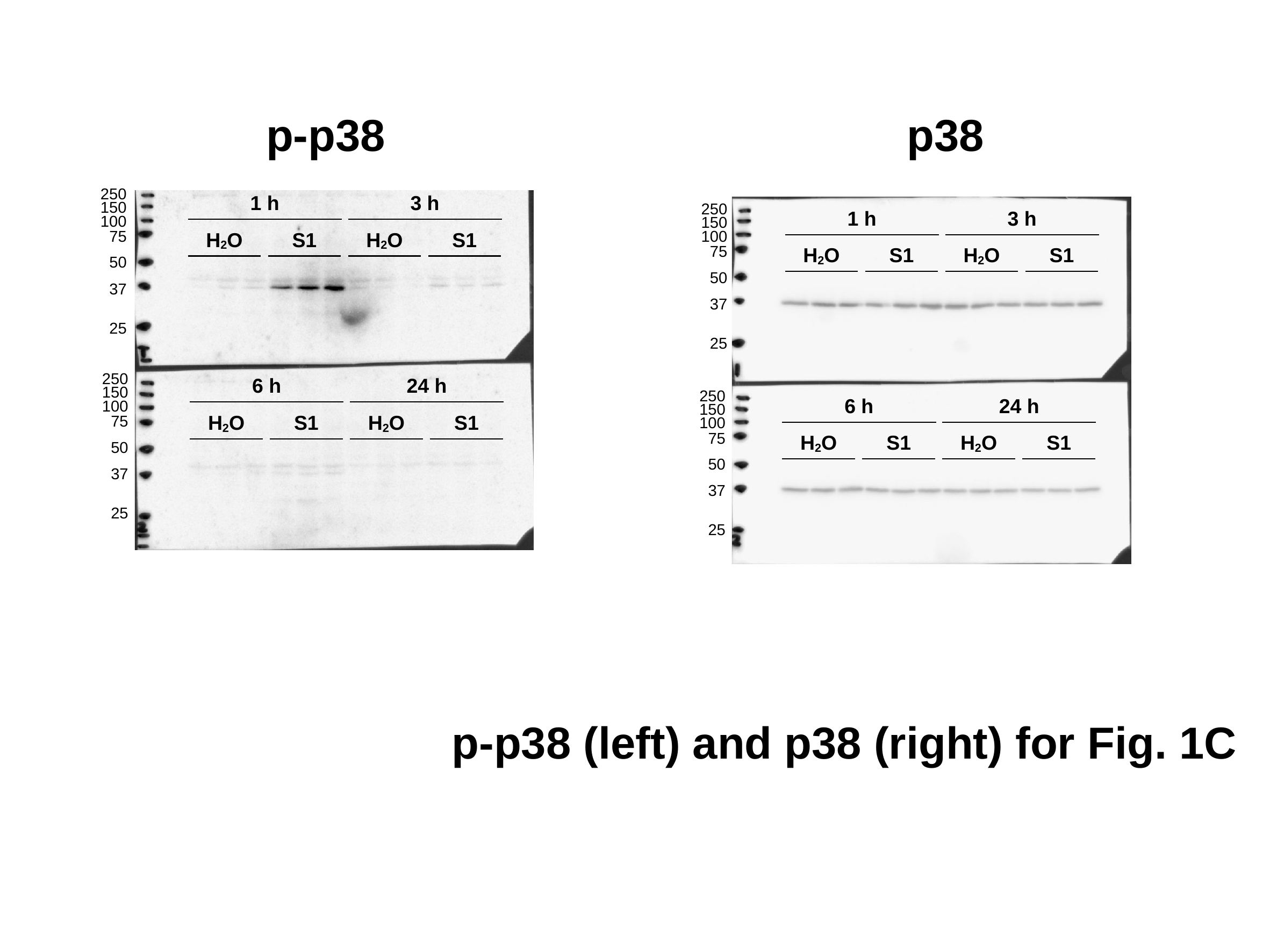

p-p38
p38
250
1 h
3 h
H2O
S1
H2O
S1
150
250
1 h
3 h
H2O
S1
H2O
S1
100
150
100
75
75
50
50
37
37
25
25
250
6 h
24 h
H2O
S1
H2O
S1
150
250
6 h
24 h
H2O
S1
H2O
S1
100
150
75
100
75
50
50
37
37
25
25
p-p38 (left) and p38 (right) for Fig. 1C

## Slide 5
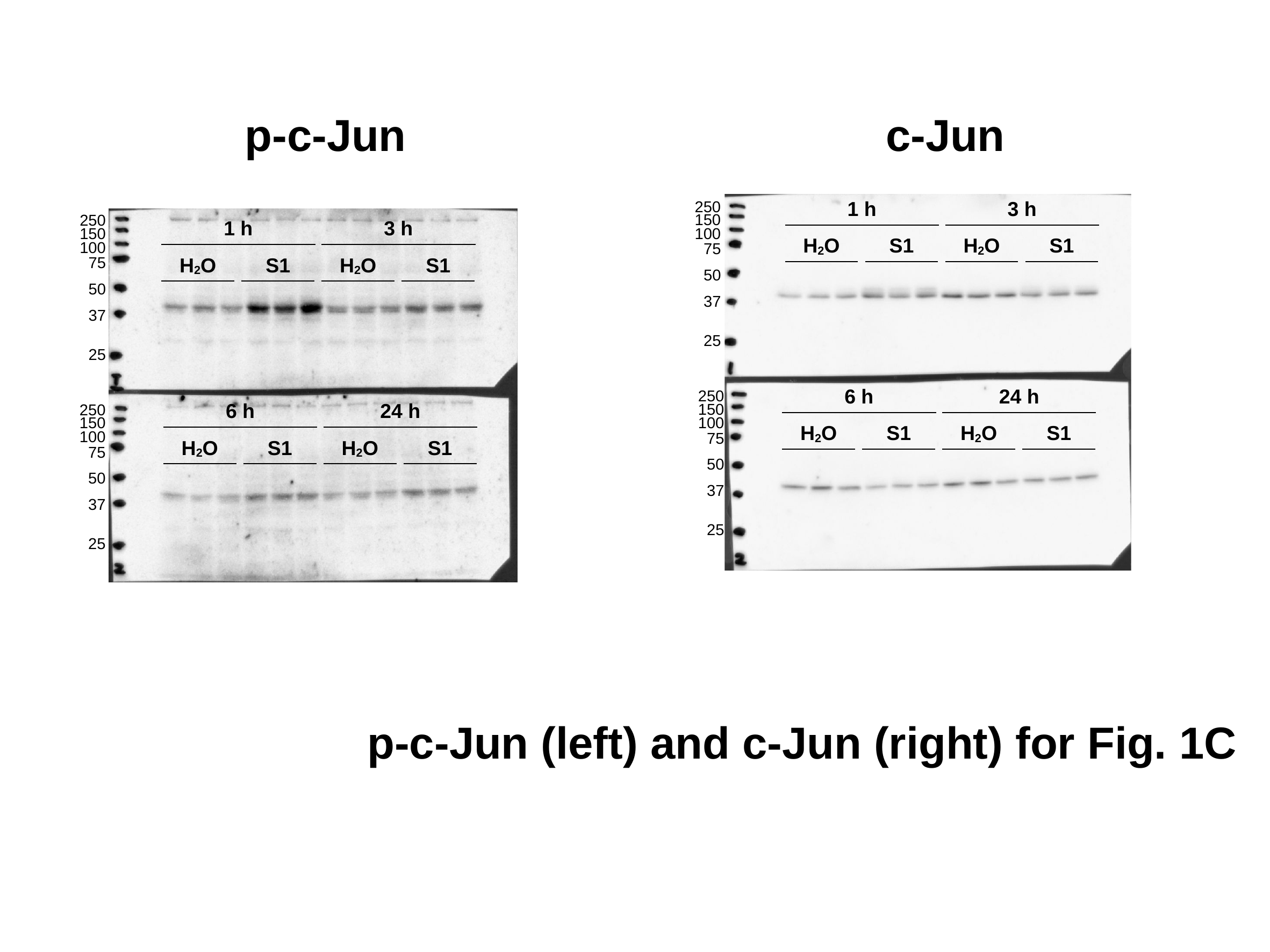

p-c-Jun
c-Jun
1 h
3 h
H2O
S1
H2O
S1
250
150
250
1 h
3 h
H2O
S1
H2O
S1
150
100
100
75
75
50
50
37
37
25
25
6 h
24 h
H2O
S1
H2O
S1
250
6 h
24 h
H2O
S1
H2O
S1
150
250
150
100
100
75
75
50
50
37
37
25
25
p-c-Jun (left) and c-Jun (right) for Fig. 1C

## Slide 6
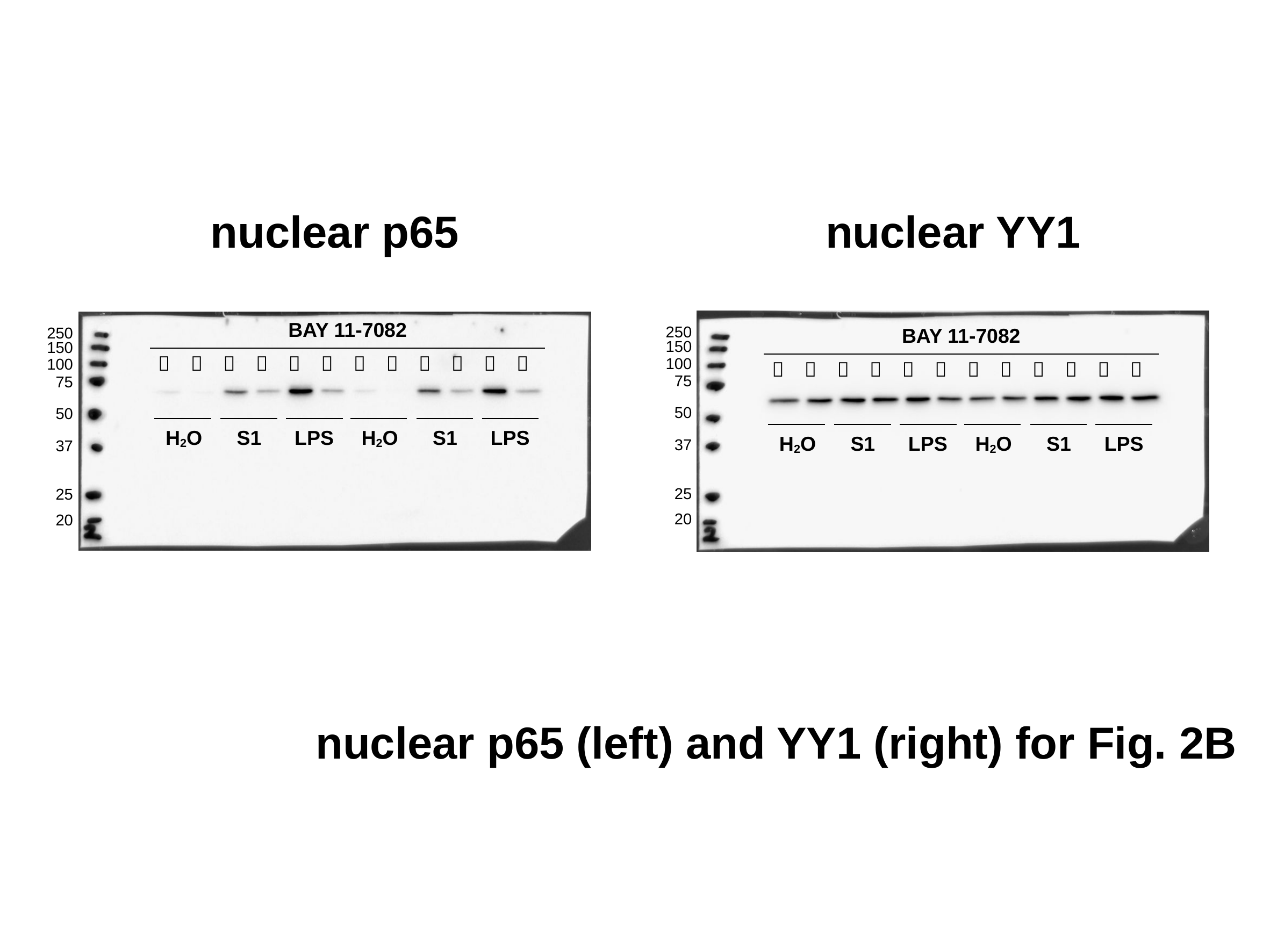

nuclear p65
nuclear YY1
BAY 11-7082
250
BAY 11-7082
250
150
150
－
＋
－
＋
－
＋
－
＋
－
＋
－
＋
100
100
－
＋
－
＋
－
＋
－
＋
－
＋
－
＋
75
75
50
50
H2O
S1
LPS
H2O
S1
LPS
H2O
S1
LPS
H2O
S1
LPS
37
37
25
25
20
20
nuclear p65 (left) and YY1 (right) for Fig. 2B

## Slide 7
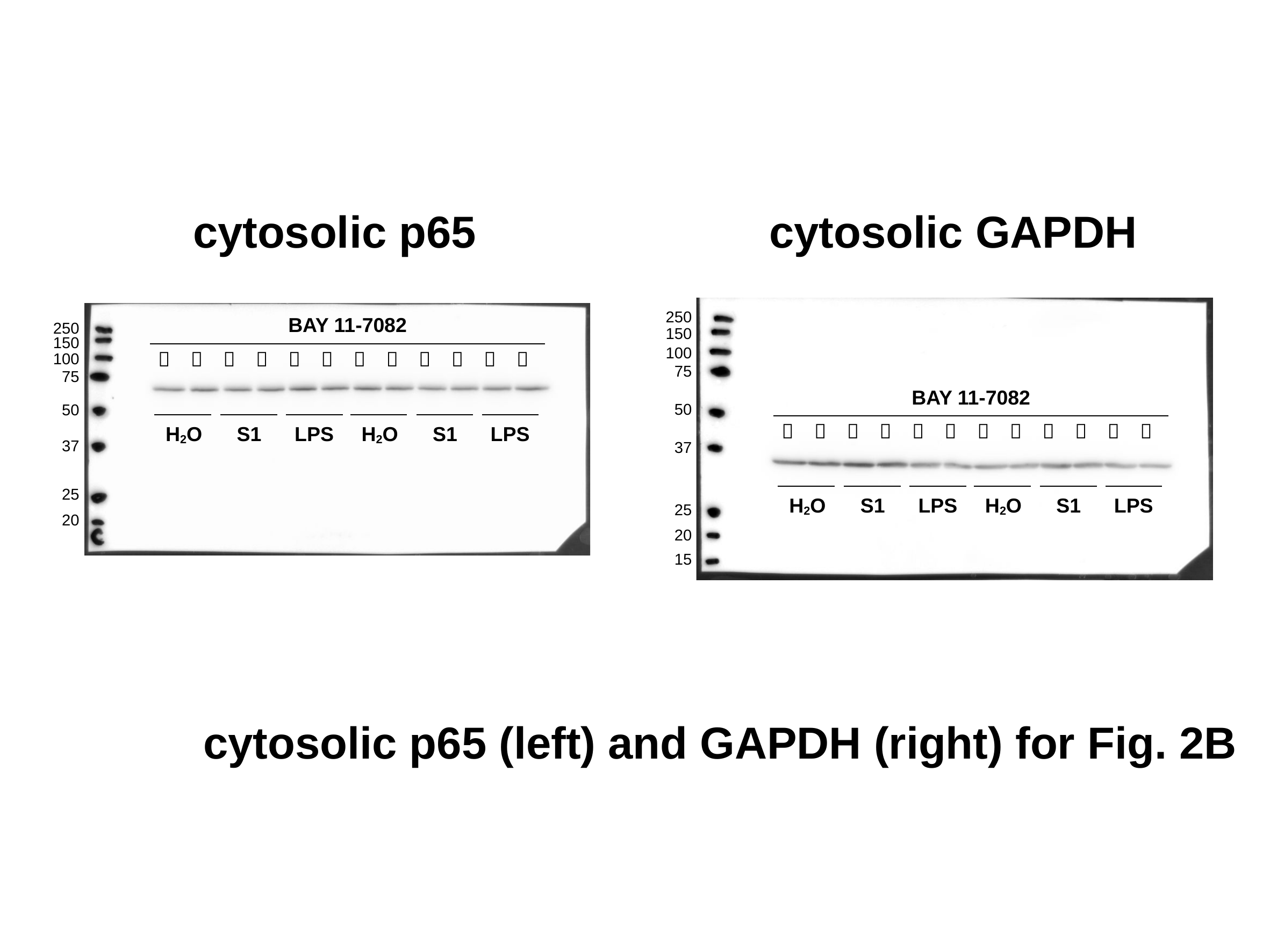

cytosolic p65
cytosolic GAPDH
250
BAY 11-7082
250
150
150
100
－
＋
－
＋
－
＋
－
＋
－
＋
－
＋
100
75
75
BAY 11-7082
50
50
－
＋
－
＋
－
＋
－
＋
－
＋
－
＋
H2O
S1
LPS
H2O
S1
LPS
37
37
25
H2O
S1
LPS
H2O
S1
LPS
25
20
20
15
cytosolic p65 (left) and GAPDH (right) for Fig. 2B

## Slide 8
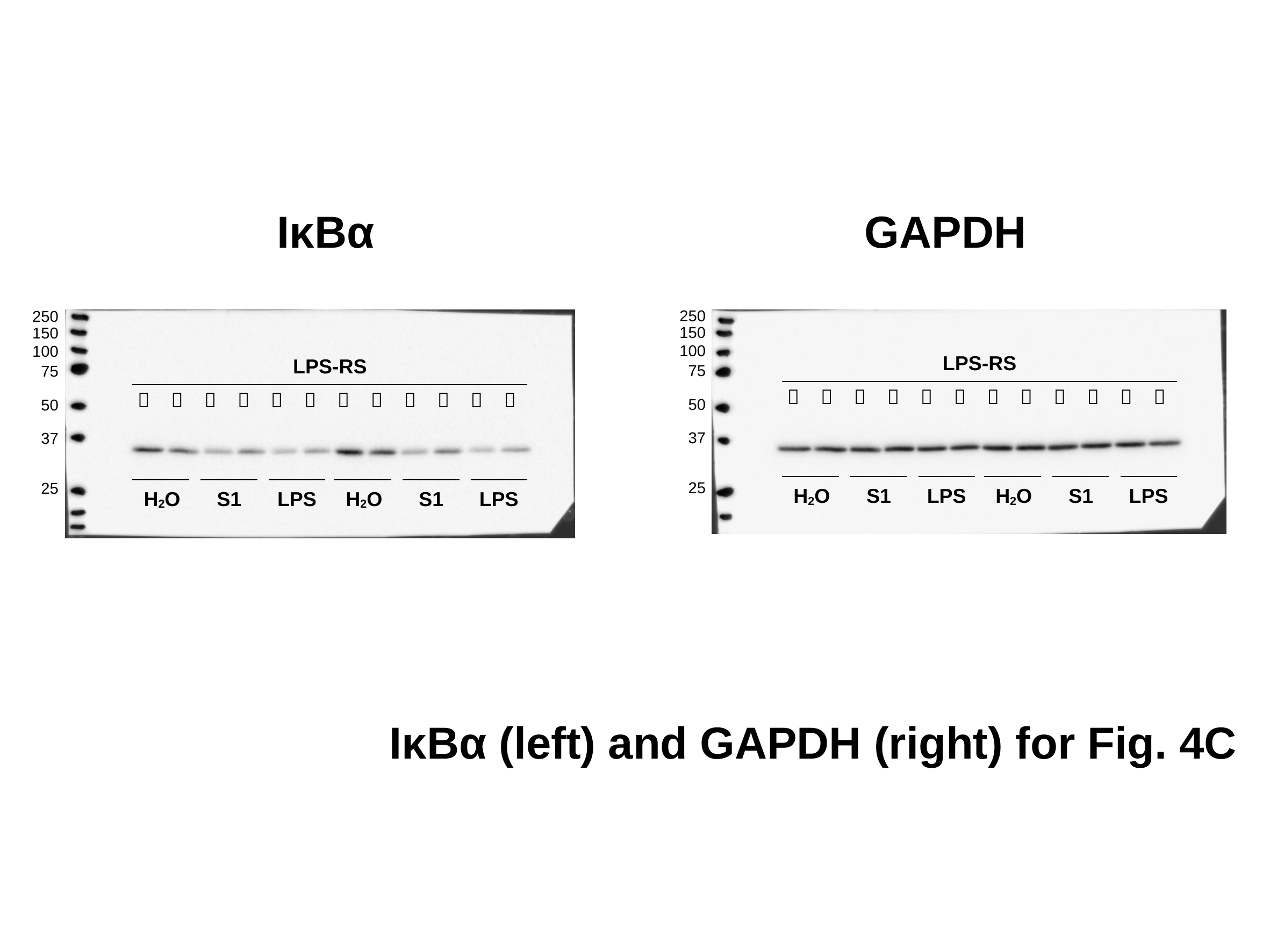

IκBα
GAPDH
250
250
150
150
100
100
LPS-RS
－
＋
－
＋
－
＋
－
＋
－
＋
－
＋
H2O
S1
LPS
H2O
S1
LPS
LPS-RS
－
＋
－
＋
－
＋
－
＋
－
＋
－
＋
H2O
S1
LPS
H2O
S1
LPS
75
75
50
50
37
37
25
25
IκBα (left) and GAPDH (right) for Fig. 4C

## Slide 9
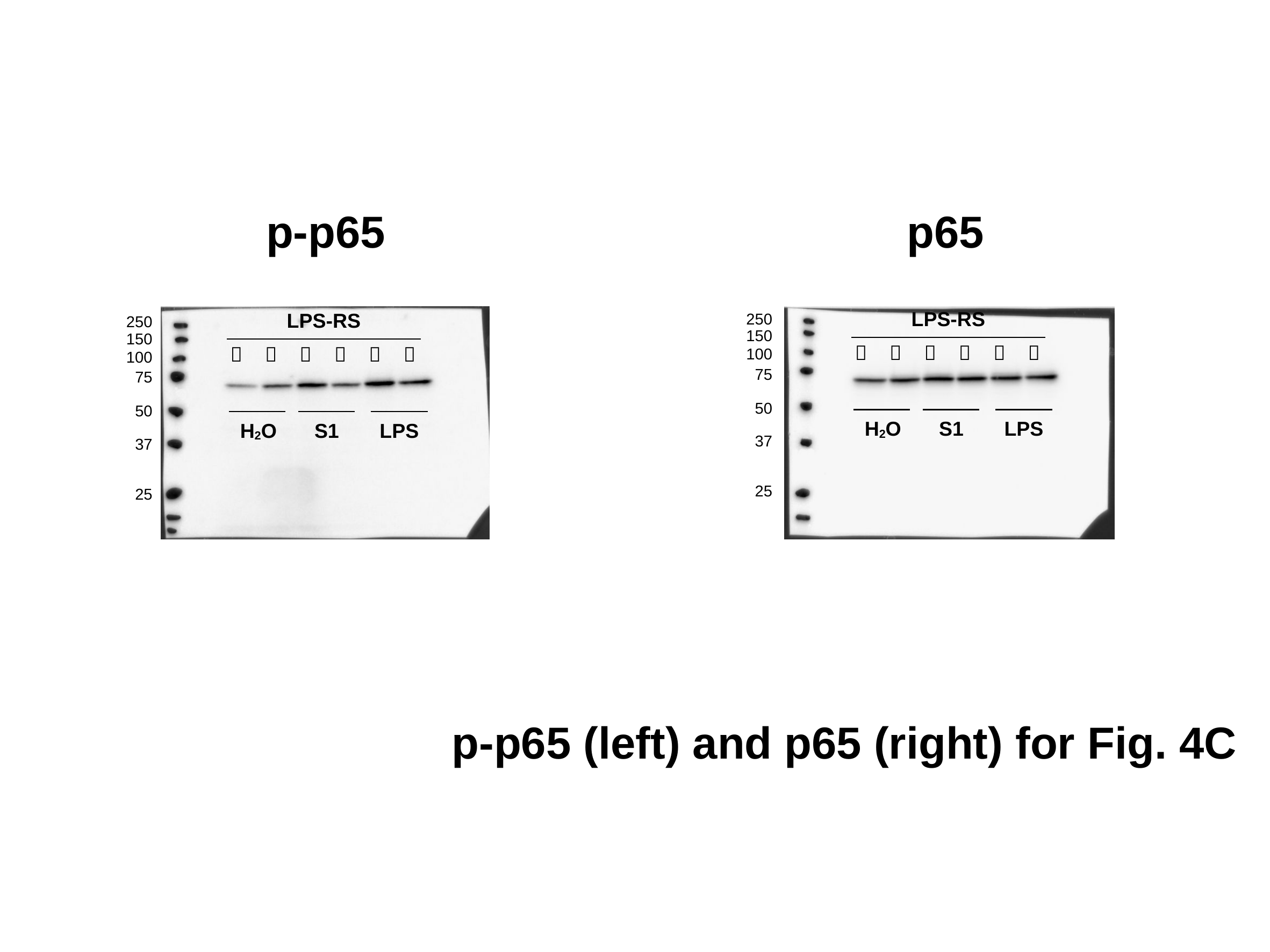

p-p65
p65
LPS-RS
LPS-RS
250
250
150
150
－
＋
－
＋
－
＋
－
＋
－
＋
－
＋
100
100
75
75
50
50
H2O
S1
LPS
H2O
S1
LPS
37
37
25
25
p-p65 (left) and p65 (right) for Fig. 4C

## Slide 10
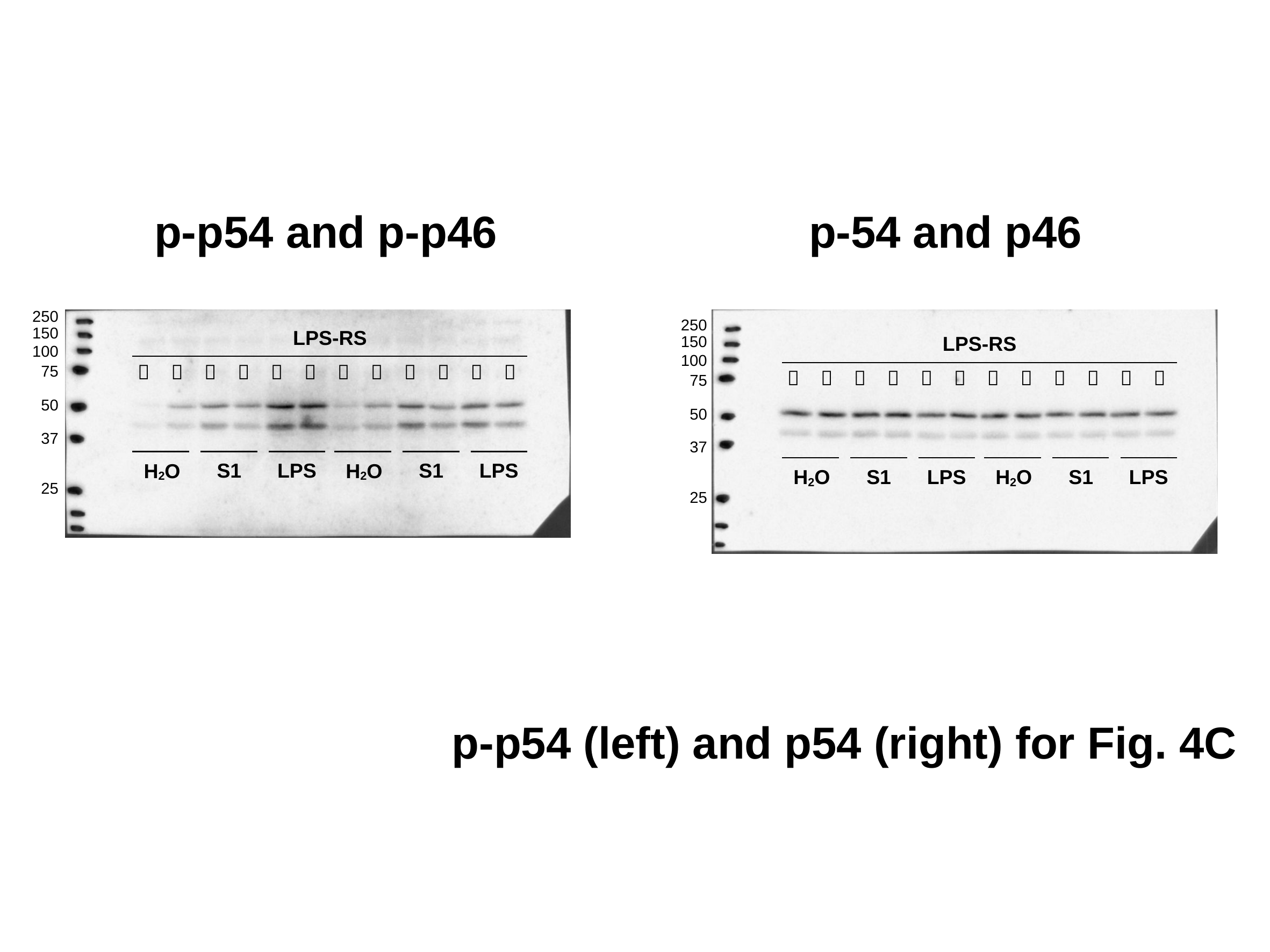

p-p54 and p-p46
p-54 and p46
250
250
150
LPS-RS
－
＋
－
＋
－
＋
－
＋
－
＋
－
＋
H2O
S1
LPS
H2O
S1
LPS
LPS-RS
－
＋
－
＋
－
＋
－
＋
－
＋
－
＋
H2O
S1
LPS
H2O
S1
LPS
150
100
100
75
75
50
50
37
37
25
25
p-p54 (left) and p54 (right) for Fig. 4C

## Slide 11
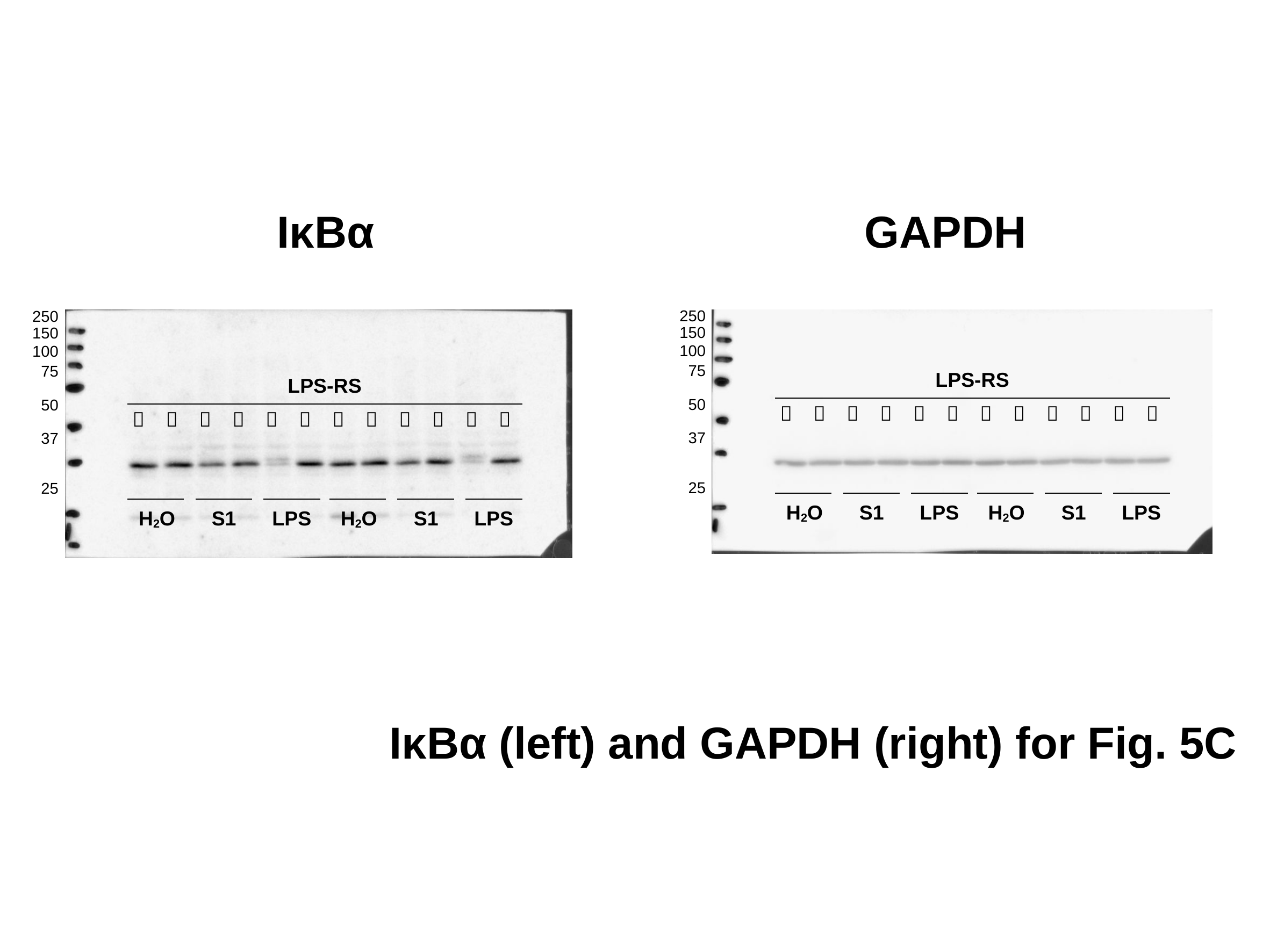

IκBα
GAPDH
250
250
150
150
100
100
75
75
LPS-RS
－
＋
－
＋
－
＋
－
＋
－
＋
－
＋
H2O
S1
LPS
H2O
S1
LPS
LPS-RS
－
＋
－
＋
－
＋
－
＋
－
＋
－
＋
H2O
S1
LPS
H2O
S1
LPS
50
50
37
37
25
25
IκBα (left) and GAPDH (right) for Fig. 5C

## Slide 12
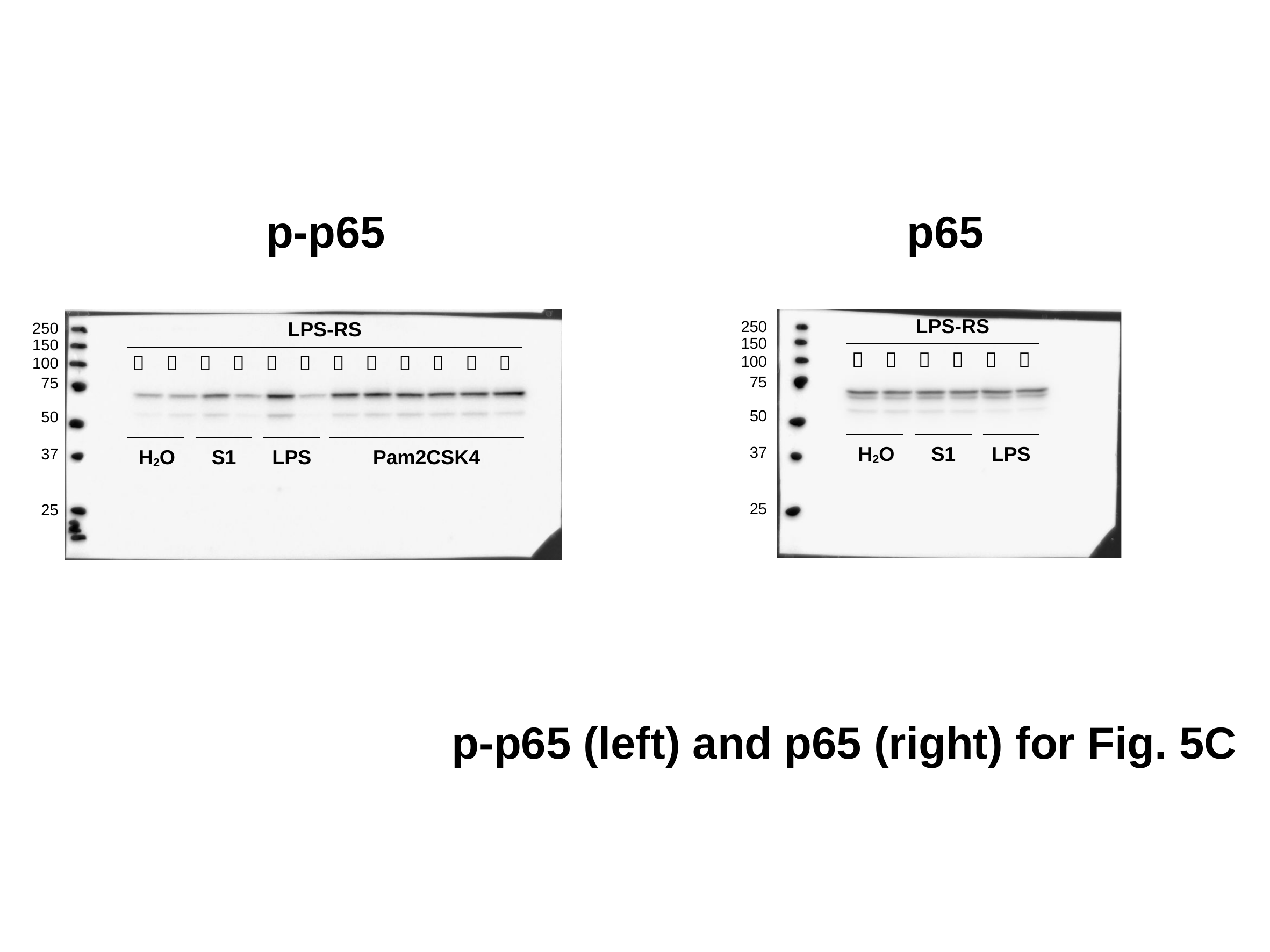

p-p65
p65
LPS-RS
LPS-RS
250
250
150
150
－
＋
－
＋
－
＋
－
＋
－
＋
－
＋
－
＋
－
＋
－
＋
100
100
75
75
50
50
H2O
S1
LPS
37
37
H2O
S1
LPS
Pam2CSK4
25
25
p-p65 (left) and p65 (right) for Fig. 5C

## Slide 13
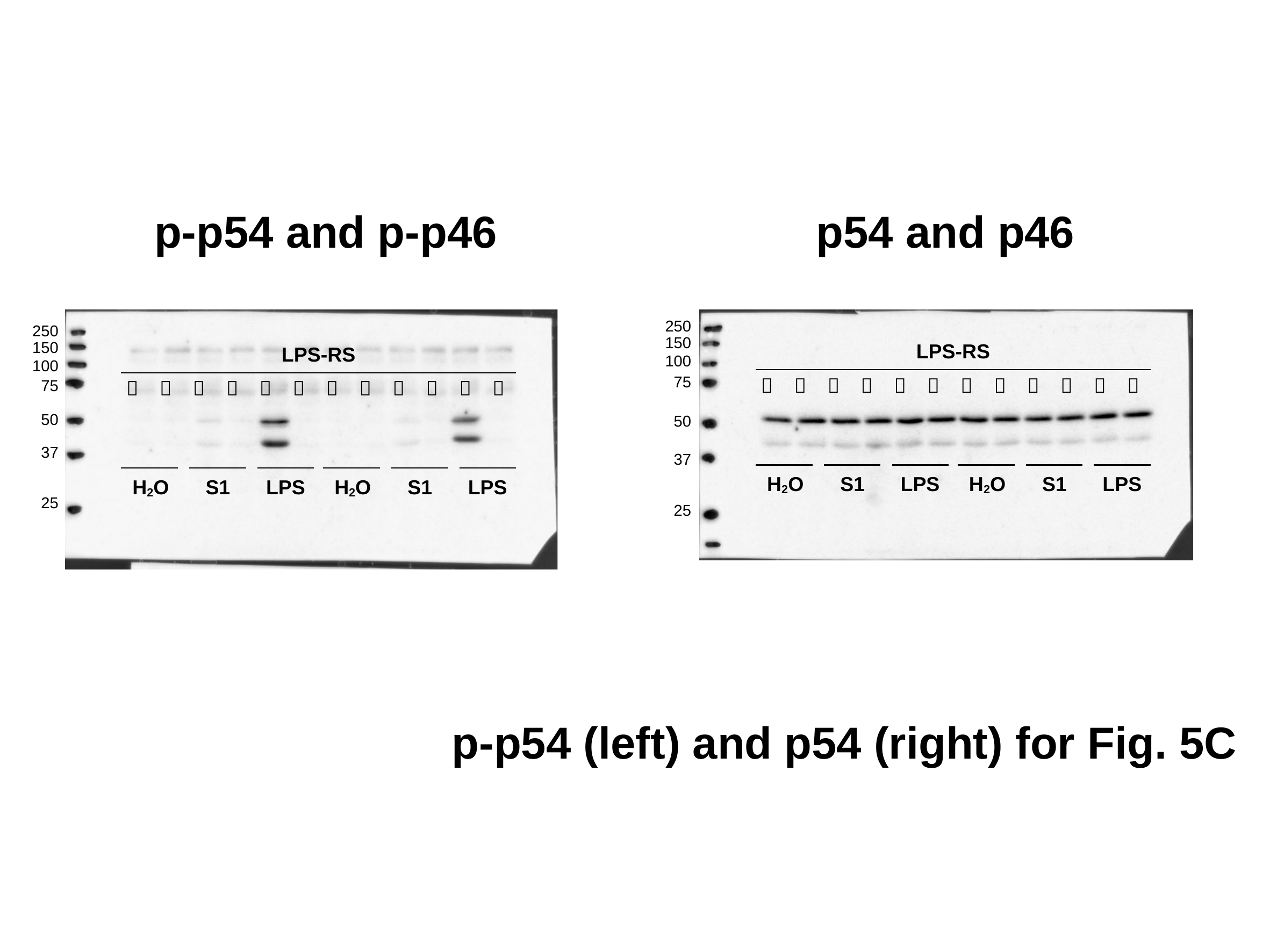

p-p54 and p-p46
p54 and p46
250
250
150
150
LPS-RS
－
＋
－
＋
－
＋
－
＋
－
＋
－
＋
H2O
S1
LPS
H2O
S1
LPS
LPS-RS
－
＋
－
＋
－
＋
－
＋
－
＋
－
＋
H2O
S1
LPS
H2O
S1
LPS
100
100
75
75
50
50
37
37
25
25
p-p54 (left) and p54 (right) for Fig. 5C

## Slide 14
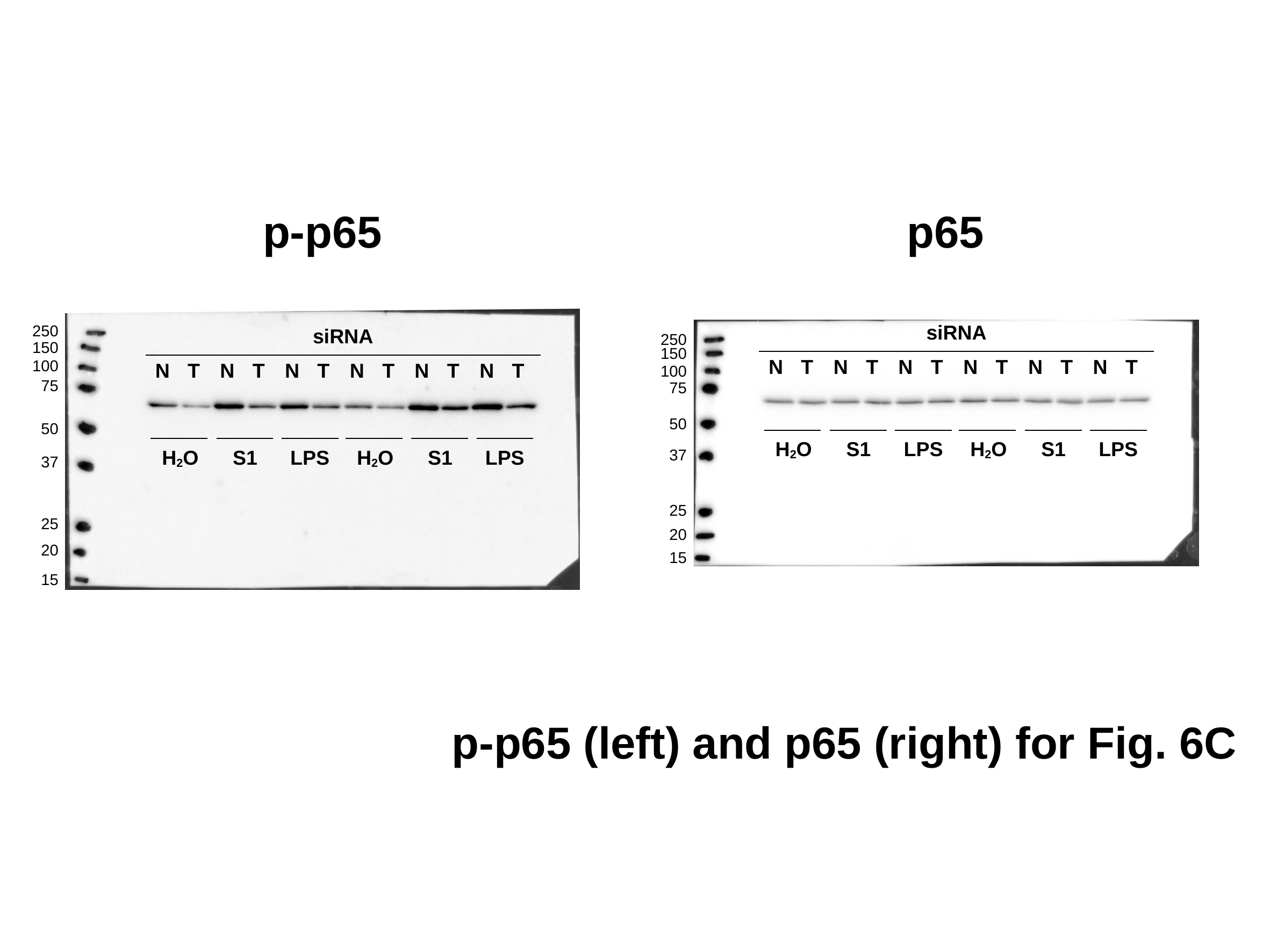

p-p65
p65
siRNA
250
siRNA
N
T
N
T
N
T
N
T
N
T
N
T
H2O
S1
LPS
H2O
S1
LPS
250
150
150
N
T
N
T
N
T
N
T
N
T
N
T
100
100
75
75
50
50
H2O
S1
LPS
H2O
S1
LPS
37
37
25
25
20
20
15
15
p-p65 (left) and p65 (right) for Fig. 6C

## Slide 15
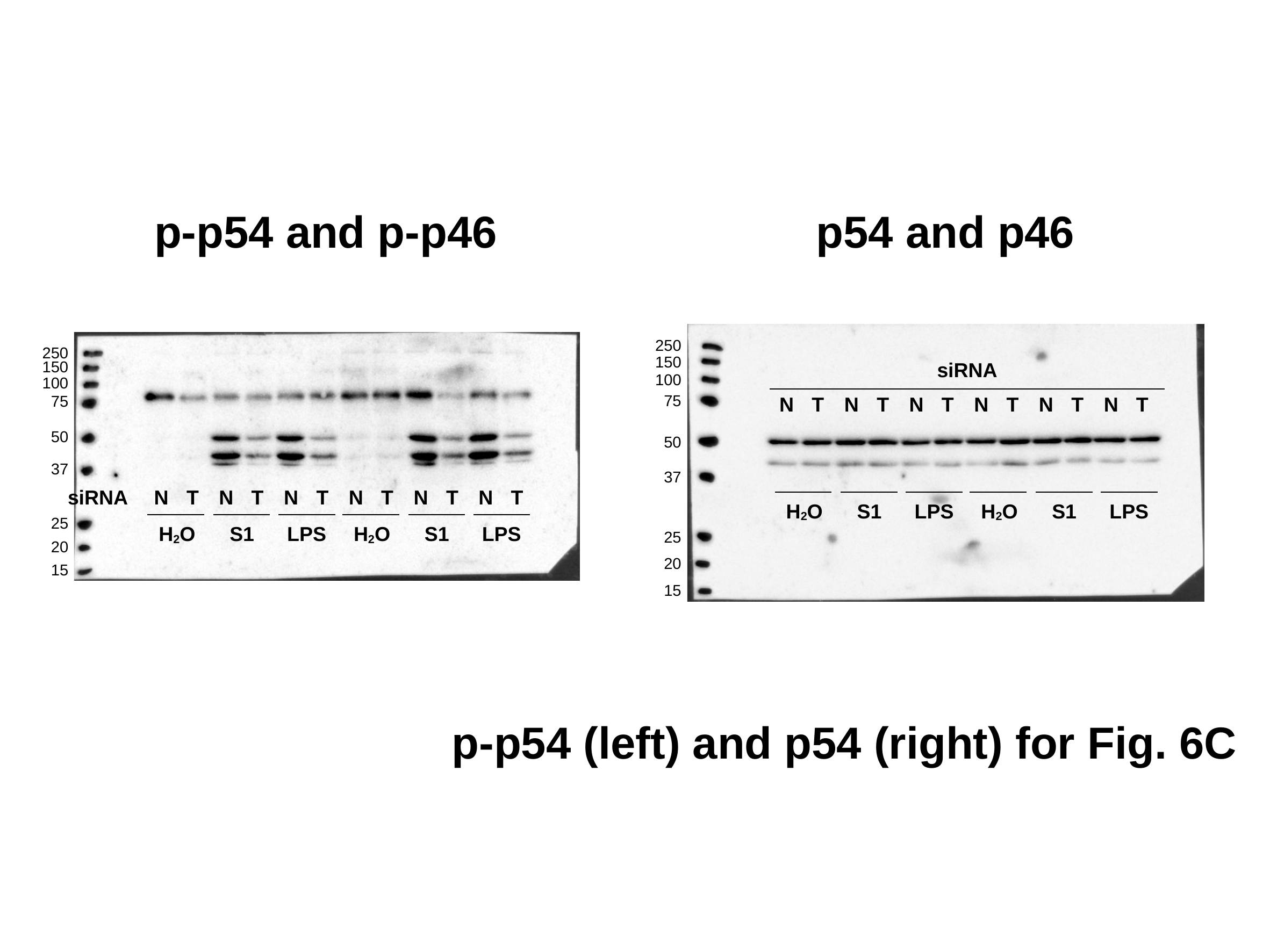

p-p54 and p-p46
p54 and p46
250
250
150
150
siRNA
100
100
75
75
N
T
N
T
N
T
N
T
N
T
N
T
50
50
37
37
siRNA
N
T
N
T
N
T
N
T
N
T
N
T
H2O
S1
LPS
H2O
S1
LPS
25
H2O
S1
LPS
H2O
S1
LPS
25
20
20
15
15
p-p54 (left) and p54 (right) for Fig. 6C

## Slide 16
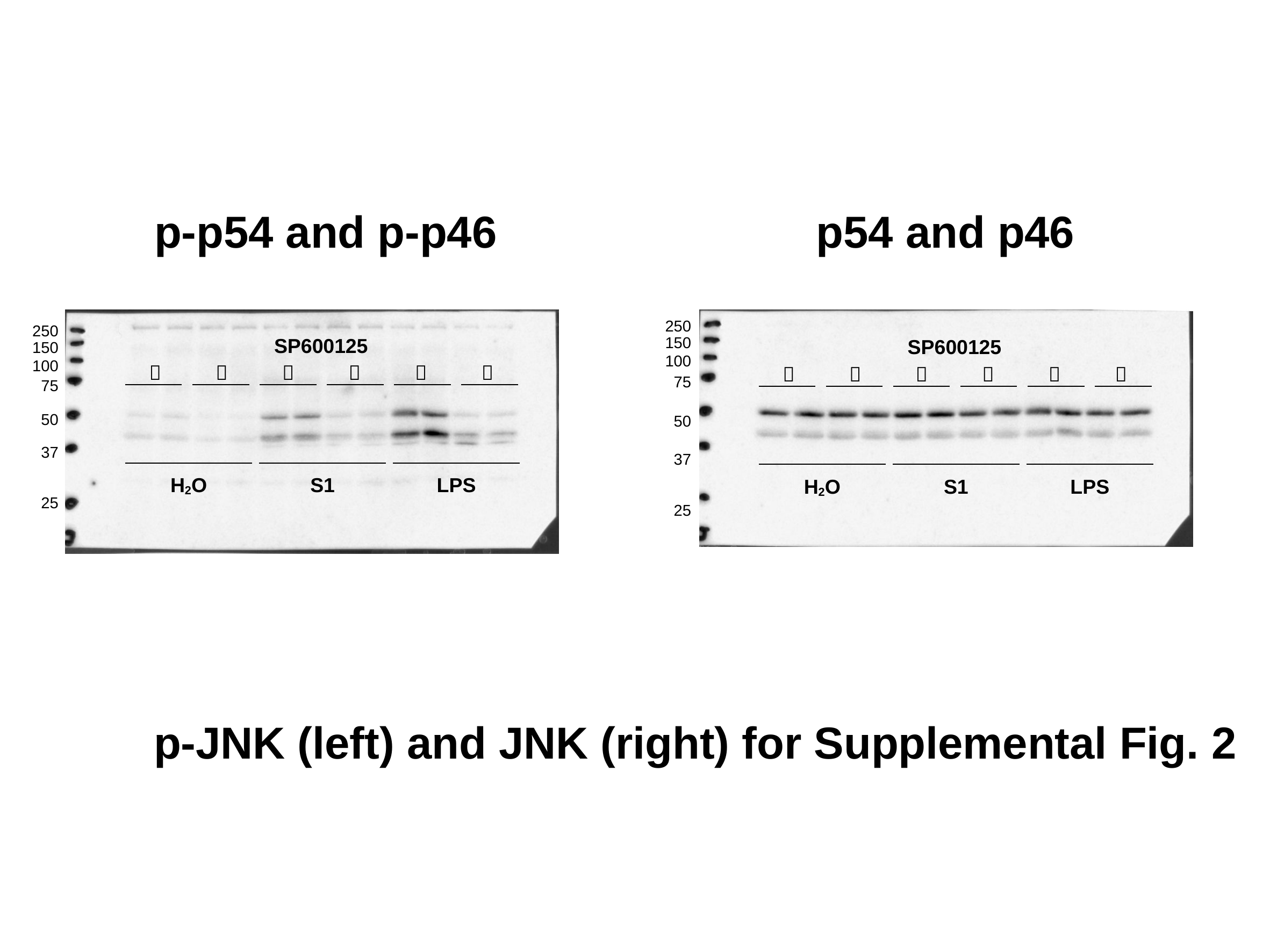

p-p54 and p-p46
p54 and p46
250
250
150
SP600125
－
＋
－
＋
－
＋
H2O
S1
LPS
SP600125
－
＋
－
＋
－
＋
H2O
S1
LPS
150
100
100
75
75
50
50
37
37
25
25
p-JNK (left) and JNK (right) for Supplemental Fig. 2

## Slide 17
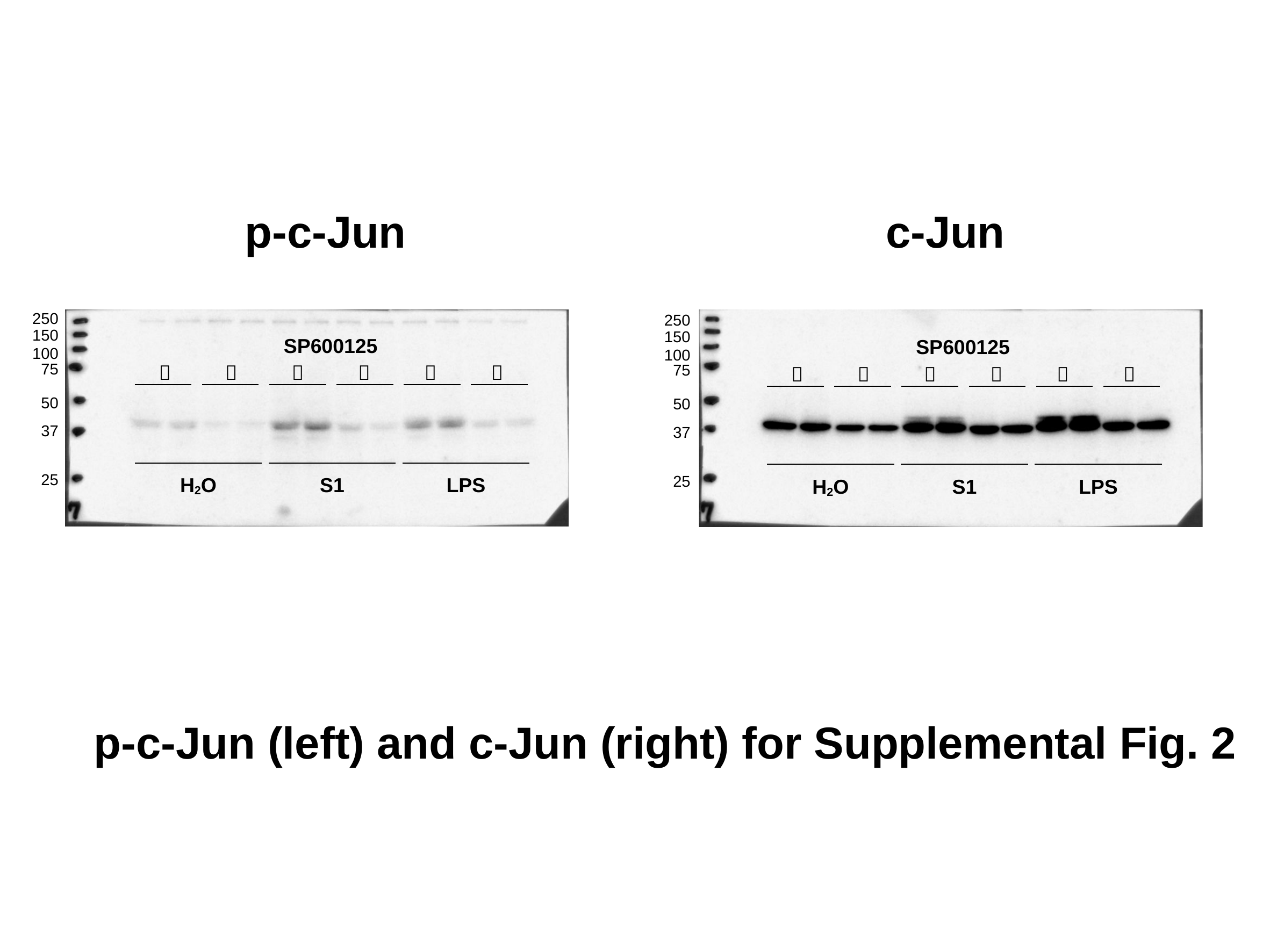

p-c-Jun
c-Jun
250
250
150
150
SP600125
－
＋
－
＋
－
＋
H2O
S1
LPS
SP600125
－
＋
－
＋
－
＋
H2O
S1
LPS
100
100
75
75
50
50
37
37
25
25
p-c-Jun (left) and c-Jun (right) for Supplemental Fig. 2
